# Risk of herpes zoster associated with JAK inhibitors in immune-mediated inflammatory diseases: a systematic review and network meta-analysis

**DOI:** 10.3389/fphar.2023.1241954

**Published:** 2023-08-08

**Authors:** Qingling Xu, Liyuan He, Yufeng Yin

**Affiliations:** ^1^ Department of Gastroenterology, Wuxi Xinwu District Xinrui Hospital, Wuxi, Jiangsu, China; ^2^ Department of Rheumatology, The First Affiliated Hospital of Soochow University, Suzhou, Jiangsu, China

**Keywords:** herpes zoster, JAK inhibitors, immune-mediated inflammatory diseases, systematic review, meta-analysis

## Abstract

**Objective:** Janus kinase (JAK) inhibitors are a novel class of drugs that have shown efficacy in treating immune-mediated inflammatory diseases (IMIDs). However, their safety profile in terms of herpes zoster infection remains unclear. We aimed to evaluate the risk of herpes zoster associated with JAK inhibitors in patients with IMIDs.

**Methods:** A systematic search of electronic databases was conducted to identify randomized controlled trials (RCTs) that evaluated the safety of JAK inhibitors in patients with IMIDs including inflammatory bowel disease (IBD), rheumatoid arthritis (RA), spondyloarthritis (SpA), psoriasis (PsO), and psoriatic arthritis (PsA). The primary outcome of interest was the incidence of herpes zoster infection. Network meta-analysis was performed to compare the risk of herpes zoster among different JAK inhibitors and placebo.

**Results:** A network meta-analysis was conducted using data from 47 RCTs including 24,142 patients. In patients with IMIDs, peficitinib 100 mg QD was associated with the highest risk of herpes zoster infection in patients with IMIDs, followed by baricitinib 4 mg QD and upadacitinib 30 mg QD. No difference in herpes zoster risk was found for other JAK inhibitors compared with placebo. Subgroup analysis indicated that higher incidence of herpes zoster was found in patients treated by baricitinib 4 mg QD, peficitinib 100 mg QD, and upadacitinib 30 mg QD only in patients with RA.

**Conclusion:** Our study suggests that some JAK inhibitors, particularly peficitinib, baricitinib, and tofacitinib, are associated with a higher risk of herpes zoster infection in patients with IMIDs.

## 1 Introduction

Immune-mediated inflammatory diseases (IMIDs) are a group of diverse conditions without known cures (McInnes and Gravallese, 2021a). Although these diseases have unique characteristics such as clinical phenotype, tissue localization, and therapeutic response profile, they also share common underlying pathogenic features such as Janus kinase (JAK)-signal transducer and activator of transcription (STAT) pathway, provide an exceptional opportunity for the application of modern molecular and computational techniques in the discovery of immunological targets and development of effective therapies (McInnes and Gravallese, 2021b; Schett et al., 2021; Rusiñol and Puig, 2023). Examples of IMIDs include broad and refractory disease spectrum including inflammatory bowel disease (IBD), rheumatoid arthritis (RA), spondyloarthritis (SpA) [including both ankylosing spondylitis (AS) and non-radiographic axial spondyloarthritis (nr-axSpA)], psoriasis (PsO), psoriatic arthritis (PsA), autoimmune thyroiditis (Hashimoto’s thyroiditis), asthma, and other immune-mediated diseases (Ortega et al., 2022; Gialouri et al., 2023). Therefore, they present significant systemic medical difficulties.

Herpes zoster, commonly known as shingles, is a viral infection caused by the reactivation of the varicella-zoster virus in individuals who have previously been infected with chickenpox (Sampathkumar et al., 2009). Herpes zoster typically presents as a painful rash, usually in a unilateral dermatome, and can result in severe complications including postherpetic neuralgia, ophthalmic involvement, and dissemination (Valladales-Restrepo et al., 2023). The incidence of herpes zoster increases with age and is further elevated in individuals with IMIDs (Harbecke et al., 2021).

JAK inhibitors are a relatively new class of immunosuppressive drugs that target intracellular signaling pathways involved in the pathogenesis of IMIDs (McInnes and Gravallese, 2021b). JAK inhibitors have demonstrated efficacy in managing IMIDs. On the other hands, their usage is linked with a higher risk of severe infections, particularly herpes zoster (Benucci et al., 2023; Yamaoka and Oku, 2023). However, the comparative risk of herpes zoster associated with different JAK inhibitors in patients with IMIDs is not well established. Some studies have suggested that certain JAK inhibitors may have a higher risk of herpes zoster than others, but these findings have not been consistently replicated across studies, and no comparison of incidence of herpes zoster were conducted in patients with IMIDs as an entity (Campanaro et al., 2021; Harkins et al., 2023; Yang et al., 2023).

The aim of this study is to systematically review and synthesize the available evidence on the risk of herpes zoster associated with JAK inhibitor therapy in patients with IMIDs, including IBD, RA, SpA, PsO, and PsA. We will also perform a network meta-analysis to compare the risk of herpes zoster across different JAK inhibitors and IMIDs.

## 2 Methods

### 2.1 Registration and ethics

This study was designed and conducted in accordance with the Preferred Reporting Items for Systematic Reviews and Meta-Analyses (PRISMA) extension statement for network meta-analyses for healthcare interventions (Hutton et al., 2015), utilizing its methods and recommendations. The study protocol was created in advance and registered in PROSPERO (CRD42023423787). All data included in the study can be found in the article and Supplementary Material and have been made openly available.

### 2.2 Search strategy

A thorough search of eligible studies was conducted by utilizing MEDLINE through PubMed, Embase, Web of science, and Cochrane Library. The search strategy incorporated medical subject heading (MeSH) terms or Emtree terms and followed the PICOS format: Population (P)—Patients with IMIDs including IBD, RA, SpA, PsO and PsA. Intervention (I)—JAK inhibitors. Comparison (C)—Placebo, and/or conventional disease-modifying agents (csDMARDs). Outcomes (O)—Incidence of herpes zoster infection. Study design (S)—Randomized placebo- or active-controlled clinical trials.

We established a timeframe from the inception of each database up to 1 May 2023, and only studies published in English were included. The search terms included “herpes zoster”, “JAK inhibitor”, “immune-mediated inflammatory diseases”, “inflammatory bowel disease”, “rheumatoid arthritis”, “spondyloarthritis”, “psoriasis”, and “psoriatic arthritis”. A detailed description of the search strategy is outlined in Supplementary Table S1. We also screened the references of the eligible studies to identify any additional studies meeting our inclusion criteria.

### 2.3 Eligibility criteria

We included phase II or III randomized controlled trials (RCTs) that met the following criteria: (1) Population: adults (age≥18 years) patients with IMIDs, including IBD, RA, SpA, PsO, and PsA; (2) Intervention: patients received treatment with a JAK inhibitor (baricitinib, decernotinib, filgotinib, ivarmacitinib, peficitinib, tofacitinib, upadacitinib), either alone or in combination with immunosuppressants; It is noteworthy that this analysis specifically focused on approved JAK inhibitors with available RCT evidence related to herpes zoster risk. Upstream kinase inhibitors could be considered in an updated analysis in the future as more data accumulates; (3) Comparator: comparisons were made between JAK inhibitor treatments and placebo or another JAK inhibitor; (4) Outcome: the incidence of herpes zoster infection associated with JAK inhibitor treatment. (5) Studies published in English.

Studies were excluded if: (1) They were pediatric trials, or included pregnant patients, patients hypersensitive to JAK inhibitors, patients with systemic disease, or those previously treated with JAK inhibitors; (2) They did not provide sufficient data to estimate odds ratios (ORs) and 95% confidence intervals (95%CIs); (3) They were reviews, lectures, comments, letter or research unable to be extracted for statistical analysis.

### 2.4 Study selection and data extraction

Two reviewers (QX and LH) independently screened the titles and abstracts of the identified papers to determine their eligibility. Full texts of the potentially eligible papers were then reviewed for inclusion. Data were extracted using a standardized data extraction form that included study characteristics, patient demographics, intervention and comparison groups, and follow-up period. Any discrepancies were resolved through discussion and consensus.

### 2.5 Quality evaluation

Two reviewers (QX and LH) independently evaluated the risk of bias of each study and any disagreements were resolved through consensus. The risk of bias for each included study was assessed using the Revised Cochrane Risk-of-Bias Tool (RoB 2) (Sterne et al., 2019). The following aspects were evaluated to determine the bias risk: randomization process, deviations from the intended interventions, missing outcome data, outcome measurement, and selection of the reported result. The certainty of evidence was classified into three levels: low risk of bias, some concerns, and high risk of bias.

### 2.6 Statistical analysis

The quantitative analysis for this network meta-analysis involved a multivariate frequentist framework which allows combining direct and indirect evidence while accounting for the correlations between the multiple treatments (Chaimani et al., 2013; Shim et al., 2017). Evidence network diagrams were constructed to clearly visualize the available direct comparisons between treatments as well as the indirect comparisons enabled through the network meta-analysis (Chaimani et al., 2013; Shim et al., 2017). The results were reported as ORs with 95%CIs. Summary ORs with 95%CIs were calculated and presented using league matrix. To predict the potential effectiveness of future trials, 95% predictive intervals (95%PrIs) of ORs were also calculated and presented using forest plots alongside the meta-analysis estimates. Surface under cumulative ranking (SUCRA) curves were used to identify the JAK inhibitors with the greatest association with herpes zoster infection. SUCRA value is expressed as a percentage ranging from 0% to 100%, with the higher the SUCRA value, the more probability achievement of endpoint. Consistency tests were conducted using Wald test (Hoaglin et al., 2011; van Valkenhoef et al., 2016). Funnel plots were drawn to evaluate any small sample effects and the publication bias of the final screening. Statistical analyses were conducted using Stata/SE (version 17.0) and R software package (version 4.2.2) with the “netmeta” and “gemtc” packages. A significance level of *p* < 0.05 was used for all statistical tests.

## 3 Results

### 3.1 Search strategy and study characteristics

**FIGURE 1:**
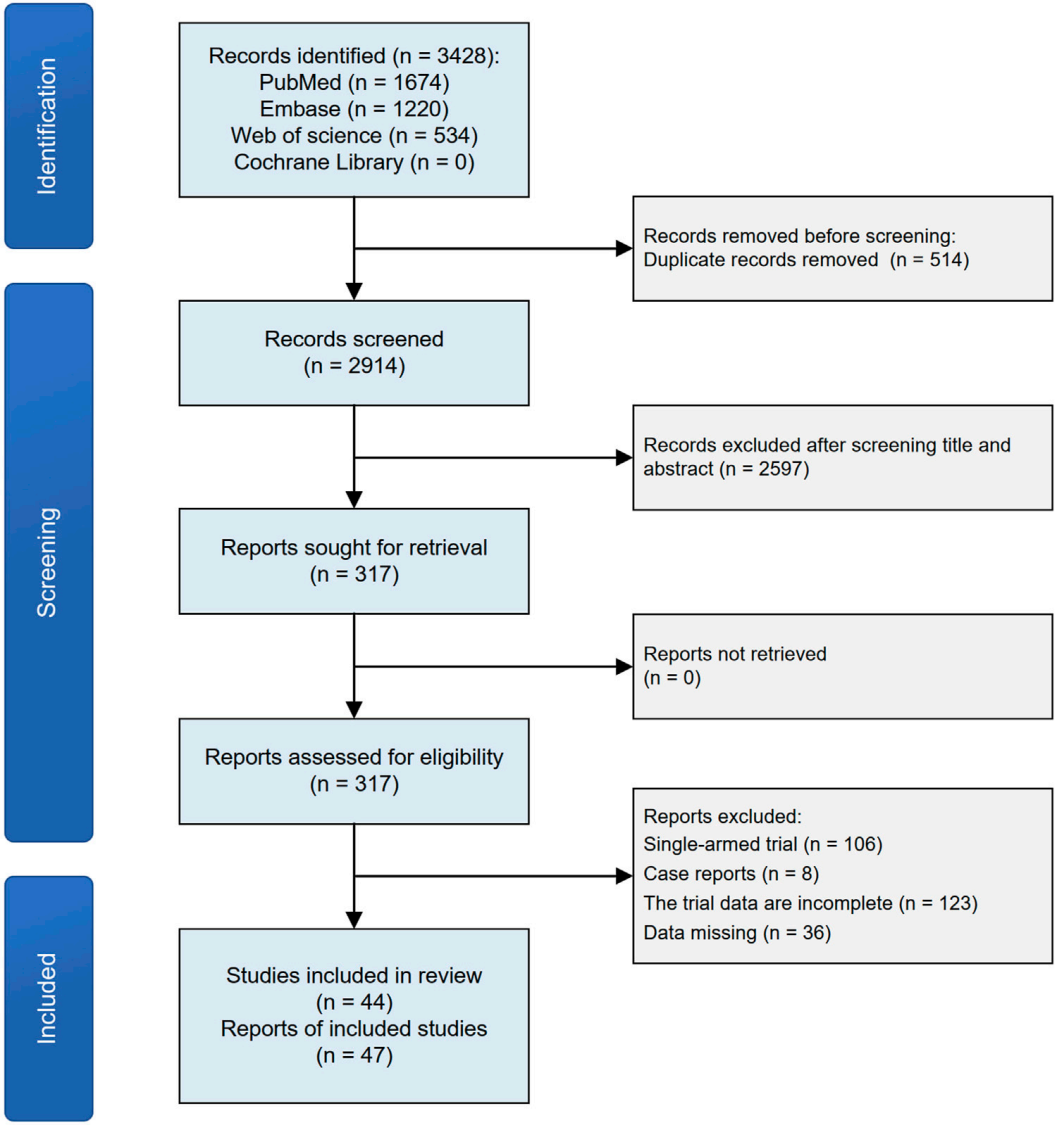
Flowchart of study selection process.

**FIGURE 2:**
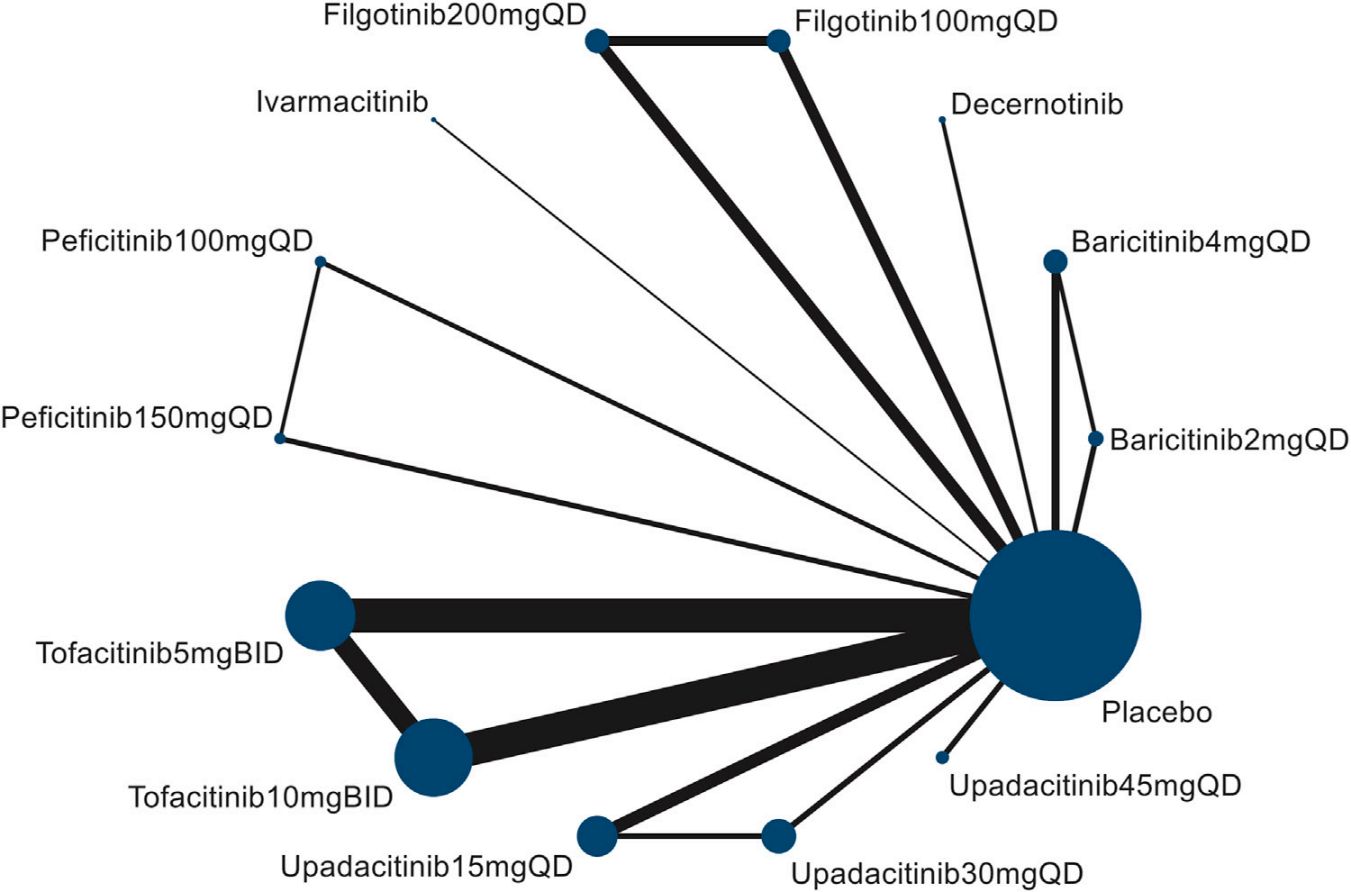
Network diagram of herpes zoster risk formed by interventions and both direct and indirect comparisons. The node size (the size of the circle) reflects the number of patients allocated to each intervention, whereas connection size (line thickness) is in proportion to each direct comparison.

The flowchart of the literature selection is shown in Figure 1. Of 3,428 studies initially identified, a total of 44 citations including 47 distinct RCTs (Fleischmann et al., 2012;van Vollenhoven et al., 2012;Kremer et al., 2013;van der Heijde et al., 2013;Lee et al., 2014;Bachelez et al., 2015;Fleischmann et al., 2015;Keystone et al., 2015;Papp et al., 2015;Tanaka et al., 2015;Genovese et al., 2016a;Genovese et al., 2016b;Takeuchi et al., 2016;Abe et al., 2017;Dougados et al., 2017;Fleischmann et al., 2017;Gladman et al., 2017;Kavanaugh et al., 2017;Kivitz et al., 2017;Mease et al., 2017;Panés et al., 2017;Sandborn et al., 2017;Taylor et al., 2017;van der Heijde et al., 2017;Vermeire et al., 2017;Westhovens et al., 2017;Zhang et al., 2017;Burmester et al., 2018;Genovese et al., 2018;Mease et al., 2018;Genovese et al., 2019;Smolen et al., 2019;Takeuchi et al., 2019;van der Heijde et al., 2019;Deodhar et al., 2021;Feagan et al., 2021;McInnes et al., 2021;Mease et al., 2021;Chen et al., 2022;Danese et al., 2022;Deodhar et al., 2022;Loftus et al., 2022;van der Heijde et al., 2022;Leng et al., 2023) were included in this network meta-analysis based on the selection criteria. The evidence network comprising 7 JAK inhibitor with 13 different dosages therapies and placebo in 4 kinds of IMIDs is shown in Figure 2 ; Supplementary Figure S1.

The characteristics of the studies included are presented in Table 1. The studies were conducted in various countries and published between 2012 and 2022. The sample sizes of the included studies ranged from 58 to 1348, with a total of 24,142 patients. The patients had a diagnosis of IMIDs, including IBD, RA, SpA (including AS and nr-axSpA), PsO, and PsA. According to the types of diseases, there were 7 studies in IBD, 25 studies in RA, 5 studies in axSpA and 4 studies in PsO, and 6 studies in PsA. The JAK inhibitors evaluated in the studies included baricitinib, decernotinib, filgotinib, ivarmacitinib, peficitinib, tofacitinib, and upadacitinib, with various dosages administered. The risk of herpes zoster was reported as ORs with 95%CIs or as incidence rates.

**TABLE 1:** Summary of the randomized controlled trials included in the meta-analysis.

Author (trial name)	Year	Phase	No. of center	Disease type	Randomised subject	JAK inhibitor regimen	Control regimen	Follow-up (week)
Inflammatory bowel disease								
Feagan (SELECTION) Feagan et al. (2021)	2021	IIb/III	341	UC	1348	Filgotinib	Placebo	10, 58
Vermeire (FITZROY) Vermeire et al. (2017)	2017	II	52	CD	174	Filgotinib	Placebo	20
Chen (AMBER2) Chen et al. (2022)	2022	II	63	UC	164	Ivarmacitinib	Placebo	8
Panes Panés et al. (2017)	2017	IIb	80	CD	180	Tofacitinib	Placebo	26
Sandborn (OCTAVE) Sandborn et al. (2017)	2017	III	144	UC	598	Tofacitinib	Placebo	52
Sandborn (OCTAVE) Sandborn et al. (2017)	2017	III	169	UC	541	Tofacitinib	Placebo	52
Danese (UC1) Danese et al. (2022)	2022	III	39	UC	474	Upadacitinib	Placebo	8
Danese (UC2) Danese et al. (2022)	2022	III	204	UC	522	Upadacitinib	Placebo	8
Loftus (U-EXCEL) Loftus et al. (2022)	2022	III	N/A	CD	526	Upadacitinib	Placebo	12
Rheumatoid arthritis								
Dougados (RA-BUILD) Dougados et al. (2017)	2017	III	182	RA	684	Baricitinib + MTX	Placebo + MTX	12, 24
Fleischmann (RA-BEGIN) Fleischmann et al. (2017)	2017	III	198	RA	588	Baricitinib + MTX	Placebo + MTX	24
Genovese (RA-BEACON) Genovese et al. (2016a)	2016	III	178	RA	527	Baricitinib + MTX	Placebo + MTX	12, 24
Keystone Keystone et al. (2015)	2015	IIb	69	RA	301	Baricitinib + MTX	Placebo + MTX	12
Taylor (RA-BEAM) Taylor et al. (2017)	2017	III	281	RA	1307	Baricitinib + MTX	Placebo + MTX	12, 24
Fleischmann and Damjanov Fleischmann et al. (2015)	2015	IIa	54	RA	206	Decernotinib + 1 DMARDs	Placebo + 1 DMARDs	12
Genovese and van Vollenhoven Genovese et al. (2016b)	2016	IIb	103	RA	359	Decernotinib + MTX	Placebo + MTX	12, 24
Genovese (FINCH 2) Genovese et al. (2019)	2019	III	114	RA	449	Filgotinib + 1–2 DMARDs	Placebo + 1–2 DMARDs	12
Kavanaugh (DARWIN 2) Kavanaugh et al. (2017)	2016	IIb	59	RA	287	Filgotinib	Placebo	12
Westhovens (DARWIN 1) Westhovens et al. (2017)	2017	IIb	106	RA	599	Filgotinib	Placebo	12, 24
Kivitz Kivitz et al. (2017)	2017	IIb	43	RA	379	Peficitinib + MTX	Placebo + MTX	12
Takeuchi (RAJ4) Takeuchi et al. (2019)	2019	III	161	RA	519	Peficitinib + MTX	Placebo + MTX	12
Takeuchi and Tanaka Takeuchi et al. (2016)	2016	IIb	43	RA	281	Peficitinib	Placebo	12
Fleischmann (ORAL Solo) Fleischmann et al. (2012)	2012	III	94	RA	611	Tofacitinib	Placebo	12
Kremer (ORAL Sync) Kremer et al. (2013)	2013	III	114	RA	795	Tofacitinib + ≥1 DMARDs	Placebo + ≥1 DMARDs	12
Lee Lee et al. (2014)	2014	III	151	RA	958	Tofacitinib	MTX	24, 48, 96
Tanaka and Takeuchi Tanaka et al. (2015)	2015	II	47	RA	318	Tofacitinib	Placebo	12
van der Heijde (ORAL Scan) van der Heijde et al. (2013)	2013	III	111	RA	797	Tofacitinib + MTX	Placebo + MTX	24
van Vollenhoven (ORAL Standard) van Vollenhoven et al. (2012)	2012	III	115	RA	717	Tofacitinib + MTX	Placebo + MTX	24
Burmester (SELECT-NEXT) Burmester et al. (2018)	2018	III	150	RA	661	Upadacitinib	Placebo	12
Genovese (SELECT-BEYOND) Genovese et al. (2018)	2018	III	153	RA	499	Upadacitinib	Placebo	12
Smolen (SELECT-MONOTHERAPY) Smolen et al. (2019)	2019	III	138	RA	648	Upadacitinib + MTX	Placebo + MTX	14
Axial spondyloarthritis								
Deodhar Deodhar et al. (2021)	2021	III	75	AS	269	Tofacitinib	Placebo	16
van der Heijde van der Heijde et al. (2017)	2017	II	58	AS	207	Tofacitinib	Placebo	12
van der Heijde (SELECT-AXIS 1) van der Heijde et al. (2019)	2019	II/III	62	AS	187	Upadacitinib	Placebo	14
van der Heijde (SELECT-AXIS 2) (AS) van der Heijde et al. (2022)	2022	III	1	AS	420	Upadacitinib	Placebo	14
Deodhar (SELECT-AXIS 2) (nr-axSpA) Deodhar et al. (2022)	2022	III	113	Nr-axSpA	313	Upadacitinib	Placebo	14
Psoriasis and psoriatic arthritis								
Abe Abe et al. (2017)	2017	III	1	PsO	58	Tofacitinib	Placebo	16
Bachelez Bachelez et al. (2015)	2015	III	112	PSO	440	Tofacitinib	Placebo	12
Papp (OPT Pivotal 1) Papp et al. (2015)	2015	III	74	PsO	900	Tofacitinib	Placebo	16
Papp (OPT Pivotal 2) Papp et al. (2015)	2015	III	94	PsO	959	Tofacitinib	Placebo	16
Zhang Zhang et al. (2017)	2017	III	1	PsO	266	Tofacitinib	Placebo	16
Mease (EQUATOR) Mease et al. (2018)	2018	II	25	PsA	191	Filgotinib	Placebo	12
Gladman Gladman et al. (2017)	2017	III	98	PsA	263	Tofacitinib	Placebo	12
Leng Leng et al. (2023)	2022	III	38	PsA	204	Tofacitinib	Placebo	12
Mease (OPAL Broaden) Mease et al. (2017)	2017	III	126	PsA	422	Tofacitinib	Placebo	12
McInnes (SELECT-PsA 1) Mcinnes et al. (2021)	2021	III	1	PsA	846	Upadacitinib	Placebo	24
Mease (SELECT-PsA 2) Mease et al. (2021)	2020	III	123	PsA	642	Upadacitinib	Placebo	24

### 3.2 Quality assessment of the included studies

The quality of all eligible studies included in this network meta-analysis was evaluated using the Jaded scale (Jadad et al., 1996). The Jaded scale ranged from 0 to 5, with 0 representing the lowest quality and 5 indicating the highest quality. Studies with a score ≥4 were considered as high quality. Common reasons for downgrading the quality of studies were: 1) not mention the method of randomization; 2) not describe the concealment of treatment allocation; 3) not blind the participant and assessor; 4) not give the reason for loss of follow up. As shown in Supplementary Table S2, the quality scores of the most included studies have very low to moderate risk of bias.

### 3.3 Major results of the network meta-analysis

**FIGURE 3:**
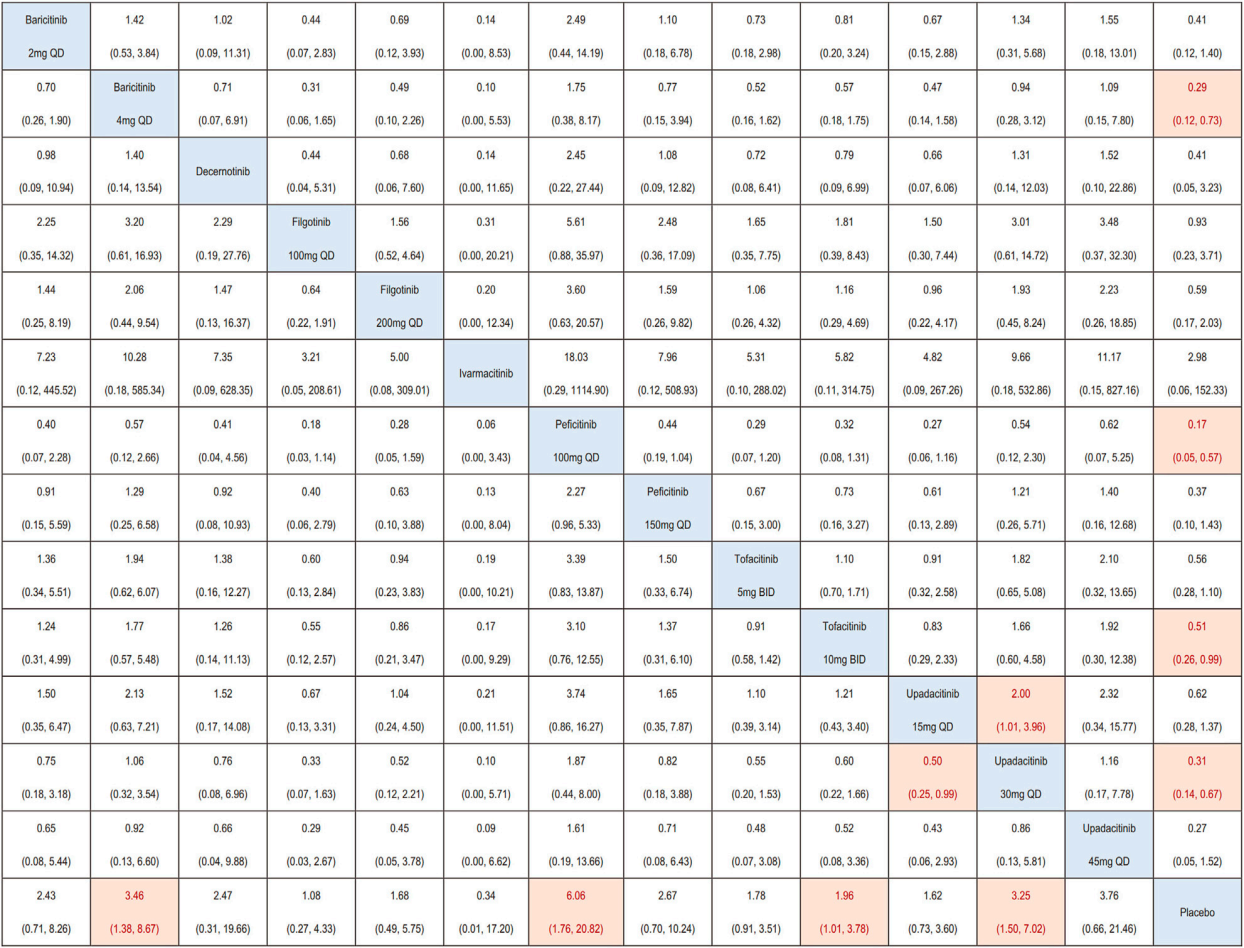
League matrix showing the comparative risk of herpes zoster included in this network meta-analysis. For interpreting the odds ratios (ORs), comparisons should be read from left to right, with reciprocals taken to obtain ORs from right to left. The ORs (95% CIs) for each comparison is shown in the cell intersecting the column-defining and row-defining treatments. Orange boxes represent statistically significant comparisons, while white boxes indicate non-statistically significant comparisons.

A total of 47 RCTs were incorporated into the network meta-analysis. In patients with IMIDs, baricitinib 4 mg QD (OR = 3.46, 95%CI 1.38, 8.67), peficitinib 100 mg QD (OR = 6.06, 95%CI 1.76, 20.82), tofacitinib 10 mg BID (OR = 1.96, 95%CI 1.01, 3.78), and upadacitinib 30 mg QD (OR = 3.25, 95%CI 1.50, 7.02) were associated with a higher incidence of herpes zoster infection compared with placebo. No difference in herpes zoster risk was found for other JAK inhibitors (baricitinib 2 mg QD, decernotinib, filgotinib, peficitinib 150 mg QD, tofacitinib 5 mg BID, upadacitinib 15 mg QD and upadacitinib 45 mg QD) compared with placebo. Besides, upadacitinib 30 mg QD (OR = 2.00, 95%CI 1.01, 3.96) had higher risk of herpes zoster compared to lower dosage of upadacitinib 15 mg QD (Figure 3).

Subgroup analysis based on different types of IMIDs indicated that, higher incidence of Herpes zoster was found in patients treated by baricitinib 4 mg QD (OR = 3.46, 95%CI 1.38, 8.67), peficitinib 100 mg QD (OR = 6.06, 95%CI 1.76, 20.82), and upadacitinib 30 mg QD (OR = 3.87, 95%CI 1.07,13.98) only in patients with RA (Supplementary Figure S2).

### 3.4 SUCRA ranking for herpes zoster risk

To provide a hierarchy of the assessed interventions, SUCRA ranking plots were generated based on the results of network meta-analysis. SUCRA values range from 0% to 100%, with higher values indicating higher hierarchy, while 50% indicates moderate risk. SUCRA plots were depicted in Supplementary Figure S3.

**FIGURE 4:**
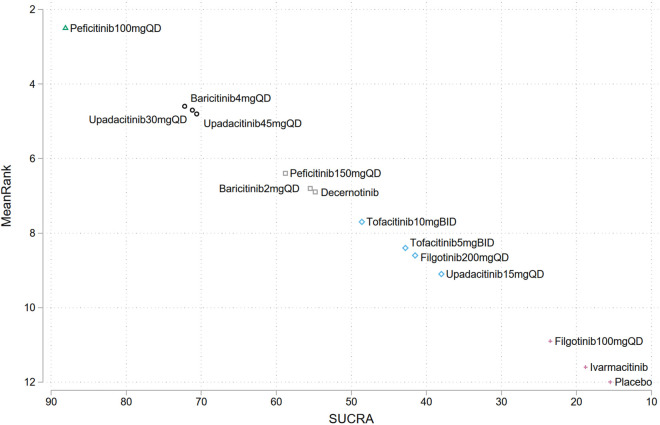
Surface under the cumulative ranking curves ranking (SUCRA) plots for the risk of herpes zoster in different JAK inhibitors. Treatments have been ranked (vertical axis) according to the SUCRA value (horizontal axis). Treatments positioned in the upper left corner of the plot have a higher risk of herpes zoster compared to the other treatments.

The SUCRA ranking plot showed that among the assessed JAK inhibitors, peficitinib 100 mg QD (SUCRA = 88.1%), were associated with the highest risk of herpes zoster infection in patients with IMIDs, followed by baricitinib 4 mg QD (SUCRA = 72.2%), upadacitinib 30 mg QD (SUCRA = 71.2%), upadacitinib 45 mg QD (SUCRA = 70.6%), peficitinib 100 mg QD (SUCRA = 58.8%), baricitinib2mgqd (SUCRA = 55.5%), and decernotinib (SUCRA = 54.8%), other JAK inhibitors dosages showed a SUCRA value less than 50%, indicating a low risk of herpes zoster infection relative to other interventions (Figure 4).

Subgroup analysis suggested a highest herpes zoster risk in patients with IBD, RA, axSpA and PsO or PsA treated with upadacitinib 45 mg QD (SUCRA = 81.2%), peficitinib100mgqd (SUCRA = 88.4%), tofacitinib10 mg BID (SUCRA = 69.6%), and tofacitinib 5 mg BID (SUCRA = 54.8%), respectively (Supplementary Figure S4).

### 3.5 Forest plot and predictive interval plot for appraising heterogeneity

The forest plots of the relative mean effects of treatments, along with 95%CIs and respective 95%PrI, were showed in (Supplementary Figure S5). The parameters of 95%PrI are crucial in effectively appraising heterogeneity among the included studies and interpreting results of the future trials by giving the range within which the results of a future study might lie (IntHout et al., 2016; Lin, 2019). Wider predictive intervals indicate more heterogeneity and uncertainty. Compared with placebo in patients with IMIDs, the 95%PrI of baricitinib 4 mg QD (0.11, 0.76) and peficitinib 100 mg QD (0.05, 0.60), and upadacitinib 30 mg QD (0.14, 0.69) exclude the null value (OR = 1), providing more confidence that these interventions are associated with higher herpes zoster risk.

Compared with placebo in patients with RA, the 95% predictive intervals of baricitinib 4 mg QD (0.09, 0.97) and peficitinib 100 mg QD (0.03, 0.83) exclude the null value, providing more confidence that these interventions are associated with higher herpes zoster risk. For other IMIDs, the 95%PrI for all comparisons crossing the null value reflect the uncertainty and inconsistency in study findings.

### 3.6 Inconsistency test

An inconsistency test was conducted to assess whether there were any significant differences between direct and indirect treatment effects, which would suggest that the studies were not consistent (Supplementary Table S3). The results of the inconsistency test showed no evidence of inconsistency in any of the analyses, indicating that the direct and indirect treatment effects were in agreement and that the studies were consistent with each other. This supports the validity of our findings and highlights the strength of our study.

### 3.7 Publication bias

Publication bias was assessed using funnel plots (Supplementary Figure S6). Based on the funnel plots generated in our network meta-analysis, the studies are expected to be symmetrically distributed at the top of the plot, which indicated the absence of publication bias. This suggests that the studies included in our analysis were not selected or published based on their results, and our findings are therefore less likely to be biased.

## 4 Discussion

The current study aimed to evaluate the risk of herpes zoster associated with JAK inhibitor therapy in patients with IMIDs including IBD, RA, SpA, PsO and PsA. The findings of this network meta-analysis suggest that there is a higher risk of herpes zoster infection in patients with RA who are treated with baricitinib 4 mg QD, peficitinib 100 mg QD, and upadacitinib 30 mg QD compared to placebo. There was no difference in herpes zoster infection risk observed in patients with RA treated with other JAK inhibitors and placebo. Besides, in patients with IBD, SpA, PsO, and PsA, no significant difference in herpes zoster infection was found with any type of JAK inhibitor or dosage compared to placebo.

The current study findings support the results of previous studies that have also reported an increased risk of herpes zoster infection with JAK inhibitors in patients with RA. A previous meta-analysis by Bechman et al. (2019) reported a significantly increased risk of herpes zoster infection with the incidence rate ratios (IRRs) of 2.86 (95% CI: 1.26, 6.50) in RA patients treated by baricitinib 4 mg QD comparing with placebo, while non-significant IRRs were seen with other JAK inhibitors such as tofacitinib and upadacitinib. A network meta-analysis also indicated that, the baricitinib 4 mg and upadacitinib 15 mg showed the highest ACR response rates; but otherwise, these two JAK inhibitors ranked higher probability in herpes zoster infection, followed by tofacitinib, adalimumab, filgotinib100 mg, and filgotinib 200 mg (Lee and Song, 2020). A meta-analysis conducted by Olivera et al. (2020) revealed an elevated risk of herpes zoster infection in patients with IMIDs (including RA, PsO, IBD and AS) who were treated with various JAK inhibitors. However, upon conducting a subgroup analysis, no significant differences were observed among the specific IMIDs. In a recent meta-analysis by Wang et al. (2022), a total of 37 RCTs with 15,174 participants treated with six different JAK inhibitors (tofacitinib, baricitinib, upadacitinib, decernotinib, peficitinib, and filgotinib) were included. The analysis revealed that only baricitinib was associated with a higher risk of herpes zoster (RR = 3.15; 95% CI: 1.19, 8.33). Our findings add to this literature by evaluating a wider range of JAK inhibitors and additional IMIDs in a network meta-analysis, allowing for more indirect comparisons between treatments.

In general populations, various risk factors such as increasing age, female gender, ethnicity of Asia and Oceania, substance abuse such as smoking or alcohol, genetic predisposition, psychological stress, and exposure to immunotoxins can collectively contribute to the elevated susceptibility to herpes zoster (Thomas and Hall, 2004; van Oorschot et al., 2021; Curran et al., 2022; Kawahira et al., 2023). The pre-existing IMIDs themselves are also associated with an increased risk of herpes zoster (Leung et al., 2022), and the use of JAK inhibitors can further increase the risk of herpes zoster in these patients (Clarke et al., 2021). We have reason to believe that the combination of these factors may lead to an even higher incidence of herpes zoster infection. However, there have been no detailed reports on the effect of the above-mentioned risk factors (age, gender, ethnicity, substance abuse et al.) on the development of herpes zoster associated with JAK inhibitors in patients with IMIDs. Besides, several phase II and III studies investigating the incidence of herpes zoster infection treated by JAK inhibitors in other IMIDs (such as atopic dermatitis, juvenile idiopathic arthritis, and non-infectious uveitis) are also in progress (Harigai and Honda, 2020). The emergence of more studies will provide more data to compare the effectiveness and safety of JAK inhibitors in different IMIDs.

The mechanism underlying the association between JAK inhibitor therapy and increased risk of herpes zoster infection is not entirely clear. It has been suggested that the activation of JAK-STAT signaling pathways is important in host defense against viral infections (Hu et al., 2021). Inhibition of JAK signaling may impair the immune system’s ability to produce antiviral cytokines such as interferons and tumor necrosis factor α, which may be a contributing factor to the increased risk of viral infections and reactivation of latent viral infections such as herpes zoster (Sunzini et al., 2020). Interestingly, even JAK inhibitors designed to target specific JAKs with high precision seem to affect the immunogenic network in overlapping ways (Moodley et al., 2016). Consequently, the precise mechanism through which certain JAK inhibitors heighten the risk of herpes zoster infection remains elusive and necessitates further investigation.

Our study found that different JAK inhibitors may be associated with different risks of herpes zoster infection in patients with RA. However, in patients with IMIDs other than RA, our study found no significant differences in the risk of herpes zoster infection among the different JAK inhibitors. In the individual studies included in our meta-analysis, the higher incidences of herpes zoster infection were found in patients with CD treated by upadacitinib 45 mg (2.9%) (Loftus et al., 2022) and PsA treated by upadacitinib 30 mg (3.7%) (Mease et al., 2021) compared with placebo. This suggests that the increased risk observed in RA patients may be specific to this population, and that caution may be warranted when prescribing certain JAK inhibitors to patients with RA. However, these results should be interpreted cautiously due to the potential limitations, including the relatively small number of studies available for analysis in some of the IMID subgroups, and the potential for heterogeneity in study design and patient populations.

## 5 Limitations

It is important to note that the results of this study should be interpreted with caution due to some limitations. Firstly, the number of studies and patients included in the analysis were limited, and some JAK inhibitors were only studied in a small number of trials. Secondly, the duration of the included studies varied, which may have affected the incidence of herpes zoster infection observed. Thirdly, the current study only included studies published in English, which may have resulted in publication bias. Finally, there were some variations in the dosages of JAK inhibitors used in the included studies, which could have influenced the results.

Despite these limitations, the findings of this study have important clinical implications. Gastroenterologist, rheumatologists, and other healthcare professionals should be aware of the increased risk of herpes zoster infection associated with certain JAK inhibitors in patients with IMIDs. Patients should be informed of this risk and monitored closely for the related signs and symptoms during treatment with JAK inhibitors. It may also be necessary to consider vaccination against herpes zoster infection in these patients.

## 6 Conclusion

In conclusion, the results of this network meta-analysis suggest that there is a higher risk of herpes zoster infection in patients with RA who are treated with baricitinib 4 mg QD, peficitinib 100 mg QD, and upadacitinib 30 mg QD compared to placebo. No higher risk of herpes zoster infection was observed in patients with other IMIDs (including IBD, SpA, PsO, and PsA) treated with JAK inhibitors. Further studies conducted in real-world settings and direct head-to-head comparisons will be required to completely understand the safety profile of the various JAK inhibitors.

## Data Availability

The original contributions presented in the study are included in the article/Supplementary Material, further inquiries can be directed to the corresponding author.
