# Supplementary material for: Risk of herpes zoster associated with JAK inhibitors in immune-mediated inflammatory diseases: a systematic review and network meta-analysis
Source: Front Pharmacol. 2023 Aug 8;14:1241954. doi: 10.3389/fphar.2023.1241954 (PMC10442487; doi:10.3389/fphar.2023.1241954)
Supplement: Supplementary file 1 [file Table1.DOCX]

Supplementary Material

Risk of Herpes zoster during Janus kinase inhibitor therapies in immune-mediated inflammatory diseases: a systematic review and network meta-analysis

Qingling Xu^1^, Liyuan He^1^, Yufeng Yin^2,*^

*** Correspondence:** Yufeng Yin: yinyufeng@126.com

## Supplementary Figures and Tables

Table S1. Search strategy

Table S2. Methodological quality and risk of bias

Figure S1. Evidence network diagram of network meta-analysis comparisons

Figure S2. League matrix of the incidence of Herpes zoster infection for all comparisons

Figure S3. SUCRA plots

Figure S4. SUCRA ranking plots

Figure S5. Forest plot with predictive interval plot for all outcomes

Table S3. Evaluation of inconsistency

Figure S6. Publication bias

## Table S1. Search strategy.

| Search date: | May 1, 2023 |
| --- | --- |
| PubMed | **Population:** (((((((((Inflammatory Bowel Diseases[Mesh]) OR (Crohn Disease[Mesh])) OR (Colitis, Ulcerative[Mesh])) OR (Arthritis, Rheumatoid[Mesh])) OR (Axial Spondyloarthritis[Mesh])) OR (Spondylitis, Ankylosing[Mesh])) OR (Non-Radiographic Axial Spondyloarthritis[Mesh])) OR (Psoriasis[Mesh])) OR (Arthritis, Psoriatic[Mesh])) AND **Intervention and Comparison:** ((((((((tofacitinib[Supplementary Concept]) OR ((((((((tofacitinib[Title/Abstract]) OR (tofacitinib citrate[Title/Abstract])) OR (Xeljanz[Title/Abstract])) OR (CP690,550[Title/Abstract])) OR (CP690550[Title/Abstract])) OR (CP-690550[Title/Abstract])) OR (CP690550[Title/Abstract])) OR (CP-690,550[Title/Abstract]))) OR (("upadacitinib" [Supplementary Concept]) OR (((Upadacitinib[Title/Abstract]) OR (ABT-494[Title/Abstract])) OR (Rinvoq[Title/Abstract])))) OR ((Peficitinib[Supplementary Concept]) OR ((Peficitinib[Title/Abstract]) OR (ASP015K[Title/Abstract])))) OR (Filgotinib[Title/Abstract])) OR (TD-1473))) OR (Janus Kinase Inhibitors[Mesh] OR "Janus Kinase Inhibitors" [Pharmacological Action])) AND **Outcome:** ("Safety" OR "Herpes Zoster" OR "Varicella-zoster Virus Infection" )  **Results: 1,674** |
| Embase | **Population:** ('immune mediated inflammatory disease'/exp OR 'rheumatoid arthritis'/exp OR 'arthritis deformans' OR 'arthritis, rheumatoid' OR 'arthrosis deformans' OR 'beauvais disease' OR 'chronic articular rheumatism' OR 'chronic polyarthritis' OR 'chronic rheumatoid arthritis' OR 'disease, beauvais' OR 'infantile rheumatoid arthritis' OR 'inflammatory arthritis' OR 'polyarthritis rheumatica' OR 'polyarthritis, primary chronic' OR 'primary chronic polyarthritis' OR 'rheumarthritis' OR 'rheumatic arthritis' OR 'rheumatic polyarthritis' OR 'rheumatism, chronic articular' OR 'rheumatoid arthritis' OR 'rheumatoid polyarthritis' OR 'axial spondyloarthritis'/exp OR 'axspa (spondyloarthritis)' OR 'axial spondylarthritis' OR 'axial spondyloarthritis' OR 'psoriatic arthritis'/exp OR 'inflammatory bowel disease'/exp OR 'inflammatory bowel disease' OR 'inflammatory bowel diseases' OR 'psoriasis'/exp OR 'psoriasiform dermatitis' OR 'psoriasiform dermatosis' OR 'psoriasiform lesion' OR 'psoriasiform rash' OR 'psoriasiform skin rash' OR 'psoriasis' OR 'psoriatic epidermis' OR 'psoriatic skin' OR 'skin rash, psoriasiform' OR 'willan lepra') AND **Intervention:** ('janus kinase inhibitor'/exp OR 'jak inhibitor' OR 'janus kinase inhibitor' OR 'janus kinase inhibitors' OR 'janus tyrosine kinase inhibitor' OR 'tofacitinib'/exp OR '1 cyanoacetyl 4 methyl n methyl n (1h pyrrolo [2, 3 d] pyrimidin 4 yl) 3 piperidinamine' OR '3 [4 methyl 3 [methyl (7h pyrrolo [2, 3 d] pyrimidin 4 yl) amino] 1 piperidinyl] 3 oxopropanenitrile' OR '3 [4 methyl 3 [methyl (7h pyrrolo [2, 3 d] pyrimidin 4 yl) amino] piperidin 1 yl] 3 oxopropanenitrile' OR '4 [n [1 (2 cyano 1 oxoethyl) 4 methyl 3 piperidinyl] n methylamino] pyrrolo [2, 3 d] pyrimidine' OR '4 methyl 3 [methyl (7h pyrrolo [2, 3 d] pyrimidin 4 yl) amino] beta oxo 1 piperidinepropanenitrile' OR 'cgb 500' OR 'cgb500' OR 'cp 690 550' OR 'cp 690, 550' OR 'cp 690550' OR 'cp 690550 10' OR 'cp 690550-10' OR 'cp690 550' OR 'cp690, 550' OR 'cp690550' OR 'cp690550 10' OR 'cp690550-10' OR 'jaquinus' OR 'pgn 600' OR 'pgn600' OR 'prd 4862257' OR 'prd4862257' OR 'ro 5169503' OR 'ro5169503' OR 'tasocitinib' OR 'tasocitinib citrate' OR 'tofacitinib' OR 'tofacitinib citrate' OR 'xeljanz' OR 'xeljanz xr' OR 'baricitinib'/exp OR '1 (ethylsulfonyl) 3 [4 (7h pyrrolo [2, 3 d] pyrimidin 4 yl) 1h pyrazol 1 yl] 3 azetidineacetonitrile' OR '[1 (ethanesulfonyl) 3 [4 (7h pyrrolo [2, 3 d] pyrimidin 4 yl) 1h pyrazol 1 yl] azetidin 3 yl] ethanenitrile' OR '[1 (ethylsulfonyl) 3 [4 (1h pyrrolo [2, 3 d] pyrimidin 4 yl) 1h pyrazol 1 yl] 3 azetidinyl] acetonitrile' OR '[1 (ethylsulfonyl) 3 [4 (7h pyrrolo [2, 3 d] pyrimidin 4 yl) 1h pyrazol 1 yl] azetidin 3 yl] ethanenitrile' OR 'baricitinib' OR 'incb 028050' OR 'incb 28050' OR 'incb028050' OR 'incb28050' OR 'ly 3009104' OR 'ly3009104' OR 'olumiant' OR 'decernotinib'/exp OR '2 methyl 2 [ [2 (1h pyrrolo [2, 3 b] pyridin 3 yl) 4 pyrimidinyl] amino] n (2, 2, 2 trifluoroethyl) butanamide' OR '2 methyl 2 [ [2 (1h pyrrolo [2, 3 b] pyridin 3 yl) pyrimidin 4 yl] amino] n (2, 2, 2 trifluoroethyl) butanamide' OR 'adelatinib' OR 'decernotinib' OR 'vrt 831509' OR 'vrt831509' OR 'vx 509' OR 'vx509' OR 'filgotinib'/exp OR 'filgotinib' OR 'filgotinib 2 butenedioate' OR 'filgotinib hydrochloride' OR 'filgotinib maleate' OR 'g 146034' OR 'g 146034 101' OR 'g 146034-101' OR 'g146034' OR 'g146034 101' OR 'g146034-101' OR 'glpg 0634' OR 'glpg0634' OR 'gs 6034' OR 'gs6034' OR 'jyseleca' OR 'n [5 [4 (1, 1 dioxothiomorpholinomethyl) phenyl] 1, 2, 4 triazolo [1, 5 a] pyridin 2 yl] cyclopropanecarboxamide' OR 'n [5 [4 (1, 1 dioxothiomorpholinomethyl) phenyl] 1, 2, 4 triazolo [1, 5 a] pyridin 2 yl] cyclopropanecarboxamide 2 butenedioate' OR 'n [5 [4 (1, 1 dioxothiomorpholinomethyl) phenyl] 1, 2, 4 triazolo [1, 5 a] pyridin 2 yl] cyclopropanecarboxamide but 2 enedioate' OR 'n [5 [4 [ (1, 1 dioxido 4 thiomorpholinyl) methyl] phenyl] 1, 2, 4 triazolo [1, 5 a] pyridin 2 yl] cyclopropanecarboxamide' OR 'n [5 [4 [ (1, 1 dioxido 4 thiomorpholinyl) methyl] phenyl] 1, 2, 4 triazolo [1, 5 a] pyridin 2 yl] cyclopropanecarboxamide 2 butenedioate' OR 'n [5 [4 [ (1, 1 dioxo 1, 4 thiazinan 4 yl) methyl] phenyl] [1, 2, 4] triazolo [1, 5 a] pyridin 2 yl] cyclopropanecarboxamide' OR 'n [5 [4 [ (1, 1 dioxothiomorpholin 4 yl) methyl] phenyl] 1, 2, 4 triazolo [1, 5 a] pyridin 2 yl] cyclopropanecarboxamide' OR 'n [5 [4 [ (1, 1 dioxothiomorpholin 4 yl) methyl] phenyl] 1, 2, 4 triazolo [1, 5 a] pyridin 2 yl] cyclopropanecarboxamide but 2 enedioate' OR 'n [5 [4 [ (1, 1 dioxothiomorpholin 4 yl) methyl] phenyl] [1, 2, 4] triazolo [1, 5 a] pyridin 2 yl] cyclopropanecarboxamide' OR 'n [5 [4 [ (1, 1 dioxothiomorpholin 4 yl) methyl] phenyl] [1, 2, 4] triazolo [1, 5 a] pyridin 2 yl] cyclopropanecarboxamide but 2 enedioate' OR 'peficitinib'/exp OR '4 [ (5 hydroxyadamantan 2 yl) amino] 1h pyrrolo [2, 3 b] pyridine 5 carboxamide' OR '4 [ (5 hydroxytricyclo [3.3.1.1 3, 7] dec 2 yl) amino] 1h pyrrolo [2, 3 b] pyridine 5 carboxamide' OR 'asp 015k' OR 'asp015k' OR 'peficitinib' OR 'peficitinib hydrobromide' OR 'upadacitinib'/exp OR '3 ethyl 4 (1, 5, 7, 10 tetrazatricyclo [7.3.0.0 (2, 6)] dodeca 2 (6), 3, 7, 9, 11 pentaen 12 yl) n (2, 2, 2 trifluoroethyl) pyrrolidine 1 carboxamide' OR '3 ethyl 4 (3h imidazo [1, 2 a] pyrrolo [2, 3 e] pyrazin 8 yl) n (2, 2, 2 trifluoroethyl) 1 pyrrolidinecarboxamide' OR '3 ethyl 4 (3h imidazo [1, 2 a] pyrrolo [2, 3 e] pyrazin 8 yl) n (2, 2, 2 trifluoroethyl) 1 pyrrolidinecarboxamide 2, 3 dihydroxybutanedioate' OR '3 ethyl 4 (3h imidazo [1, 2 a] pyrrolo [2, 3 e] pyrazin 8 yl) n (2, 2, 2 trifluoroethyl) 1 pyrrolidinecarboxamide tartrate' OR '3 ethyl 4 (3h imidazo [1, 2 a] pyrrolo [2, 3 e] pyrazin 8 yl) n (2, 2, 2 trifluoroethyl) pyrrolidine 1 carboxamide' OR '3 ethyl 4 (3h imidazo [1, 2 a] pyrrolo [2, 3 e] pyrazin 8 yl) n (2, 2, 2 trifluoroethyl) pyrrolidine 1 carboxamide 2, 3 dihydroxybutanedioate' OR '3 ethyl 4 (3h imidazo [1, 2 a] pyrrolo [2, 3 e] pyrazin 8 yl) n (2, 2, 2 trifluoroethyl) pyrrolidine 1 carboxamide tartrate' OR 'abt 494' OR 'abt494' OR 'rinvoq' OR 'upadacitinib' OR 'upadacitinib 2, 3 dihydroxybutanedioate' OR 'upadacitinib hemihydrate' OR 'upadacitinib hydrate' OR 'upadacitinib tartrate' OR 'ivarmacitinib'/exp OR 'arq 252' OR 'arq252' OR 'hexahydro n (3 methoxy 1, 2, 4 thiadiazol 5 yl) 5 (methyl 7h pyrrolo [2, 3 d] pyrimidin 4 ylamino) cyclopenta [c] pyrrole 2 (1h) carboxamide' OR 'ivarmacitinib' OR 'ivarmacitinib sulfate' OR 'n (3 methoxy 1, 2, 4 thiadiazol 5 yl) 5 [methyl (7h pyrrolo [2, 3 d] pyrimidin 4 yl) amino] hexahydrocyclopenta [c] pyrrole 2 (1h) carboxamide' OR 'shr 0302' OR 'shr0302') AND **Comparison:** ('placebo'/exp OR 'placebo' OR 'placebo gel' OR 'placebos' OR 'disease modifying antirheumatic drug'/exp OR 'disease modifying antirheumatic agent' OR 'disease modifying antirheumatic drug' OR 'disease modifying antirheumatic drugs') AND **Outcomes:** ('herpes zoster'/exp OR 'varicella zoster virus infection' OR 'varicellovirus infection' OR 'disseminated herpes zoster' OR 'herpes zoster' OR 'herpes zoster infection' OR 'herpes zoster neuralgia' OR 'herpes zoster paralysis' OR 'shingles' OR 'varicella zoster infection' OR 'zoster' OR 'zoster, herpes' OR 'virus infection'/exp OR 'disease, viral' OR 'infection, viral' OR 'infection, virus' OR 'tumor virus infections' OR 'tumour virus infections' OR 'viral disease' OR 'viral infection' OR 'virus disease' OR 'virus diseases' OR 'virus infection' OR 'infection'/exp OR 'accidental infection' OR 'acute infection' OR 'autoinfection' OR 'bacterial infections and mycoses' OR 'bacteroid infection' OR 'chain of infection' OR 'focal infection' OR 'infection' OR 'infection mechanism' OR 'infection route' OR 'infection, focal' OR 'infections' OR 'infectious disease' OR 'infectivity' OR 'route of infection') AND **Study design:** ('randomized controlled trial'/exp OR 'controlled trial, randomized' OR 'randomised controlled study' OR 'randomised controlled trial' OR 'randomized controlled study' OR 'randomized controlled trial' OR 'trial, randomized controlled' OR 'clinical study'/exp OR 'clinical data' OR 'clinical studies as topic' OR 'clinical study' OR 'medical trial')  **Results: 1,220** |
| Web of science | #1 **Intervention:** Results for Janus kinase inhibitor (Topic) OR JAK inhibitor (Topic) OR Ivarmacitinib (Topic) OR Baricitinib (Topic) OR Decernotinib (Topic) OR Filgotinib (Topic) OR Peficitinib (Topic) OR Tofacitinib (Topic) OR Upadacitinib (Topic) OR Xeljanz (Topic) OR Xeljanz (Topic) OR CP690550 (Topic) OR CP-610550 (Topic) OR CP 610550 (Topic) OR CP-690,550 (Topic) and Preprint Citation Index (Exclude – Database)  **Results: 4,791**  #2 **Population and Comparison:** Results for crohn disease (Topic) OR Ulcerative Colitis (Topic) OR Idiopathic Proctocolitis (Topic) OR Colitis Gravis (Topic) OR Inflammatory Bowel Disease (Topic) OR Colitis, Ulcerative (Topic) OR rheumatoid arthritis (Topic) OR axial spondyloarthritis (Topic) OR spondyloarthritis (Topic) OR psoriatic arthritis (Topic) OR psoriasis (Topic) OR ankylosing spondylitis (Topic) OR immune-mediated inflammatory disease (Topic) and Preprint Citation Index (Exclude – Database)  **Results: 661,350**  #3 **Outcome:** Herpes zoster (Topic)  **Results: 31,937**  #4 #1 AND #2 AND #3  **Results: 534** |
| Cochrane Library | **'Population** ( "Inflammatory Disorder Of Immune System" OR "Inflammatory Bowel Disease" OR "Rheumatoid Arthritis" OR "Ulcerative Colitis" OR "Crohn's Disease" OR "Psoriatic Arthritis" OR "Psoriasis" ) AND **Intervention** "Janus kinase inhibitor" AND **Comparison** ( "Placebo" OR "Disease-Modifying Antirheumatic Drug" ) AND **Outcome** ( "Safety Finding" OR "Herpes Zoster" OR "Varicella-zoster Virus Infection" )'  **Results: 0** |

## Table S2. Methodological quality and risk of bias


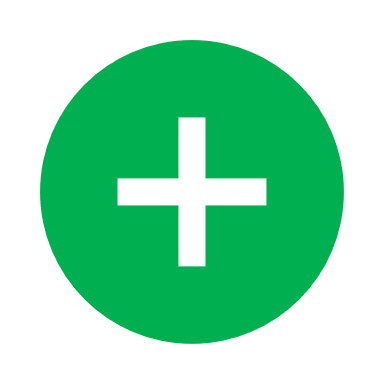
Low risk of bias
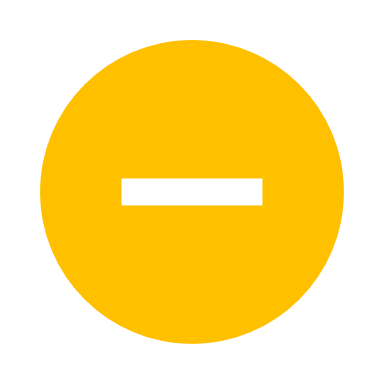
Some concerns
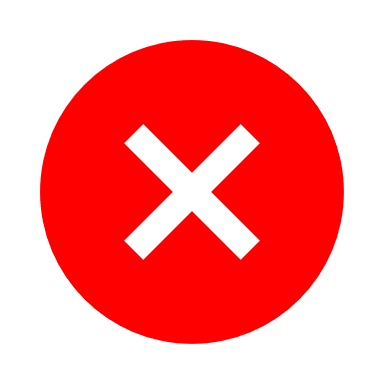
High risk of bias

| **Author (trial name)** | Randomisation process | Deviations from the intended intervention | Missing outcome data | Measurement of the outcome |
| --- | --- | --- | --- | --- |
| Inflammatory bowel disease |  |  |  |  |
| Feagan (SELECTION)A (Feagan et al., 2021) | 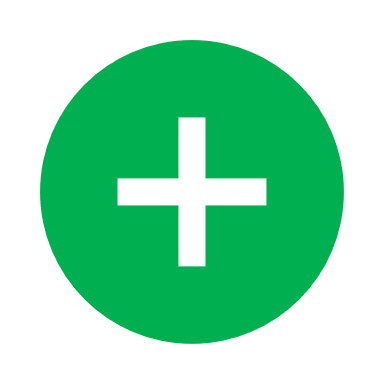 | 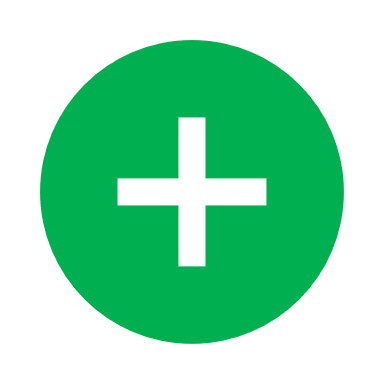 | 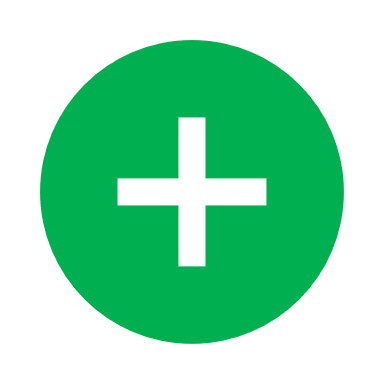 | 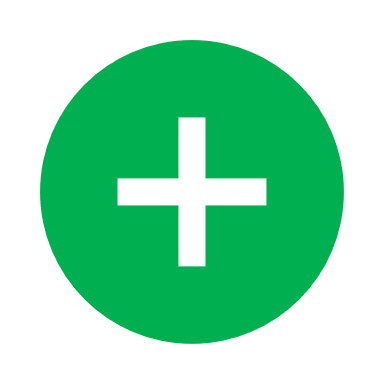 |
| Vermeire (FITZROY) (Vermeire et al., 2017) | 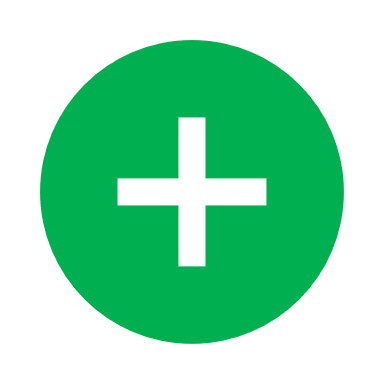 | 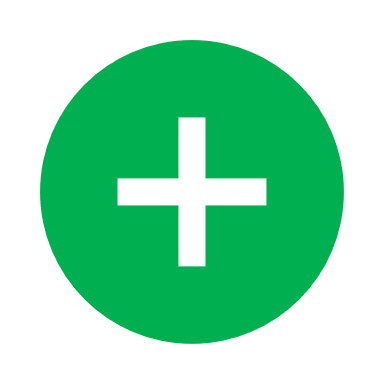 | 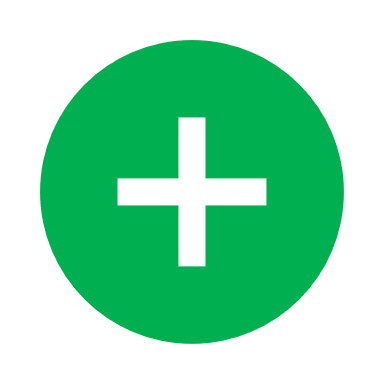 | 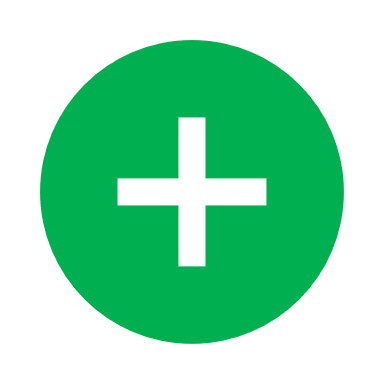 |
| Chen (AMBER2) (Chen et al., 2022) | 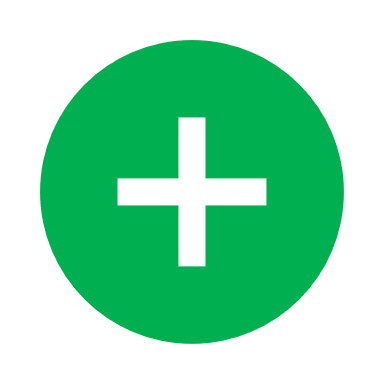 | 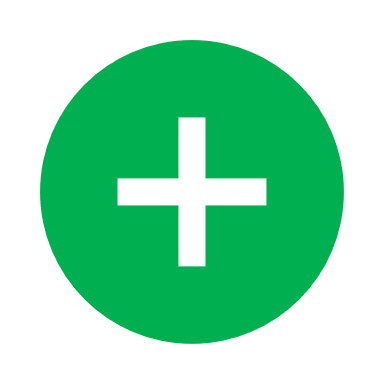 | 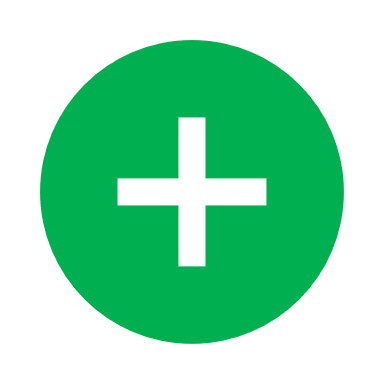 | 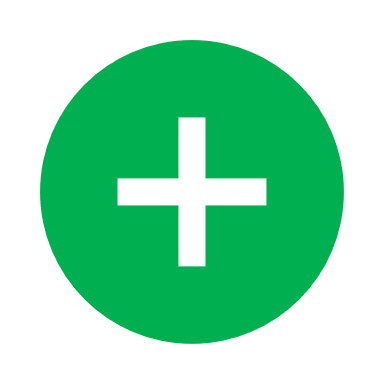 |
| Panes (Panés et al., 2017) | 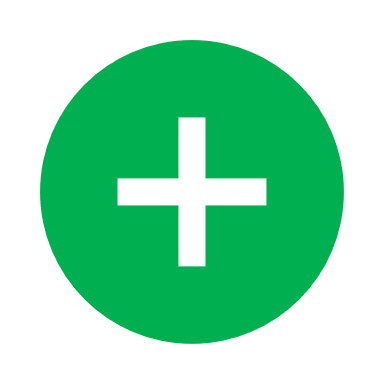 | 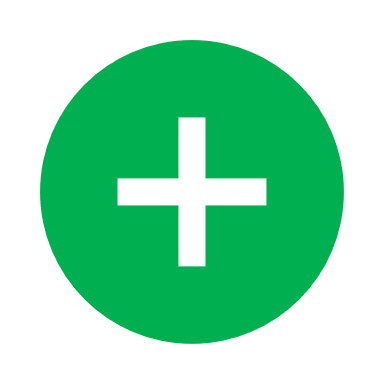 | 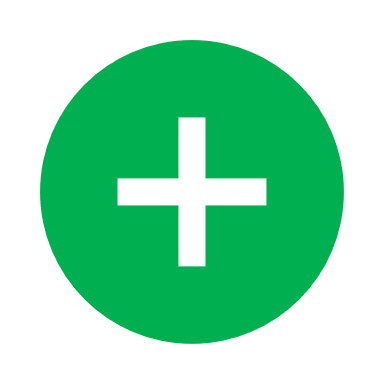 | 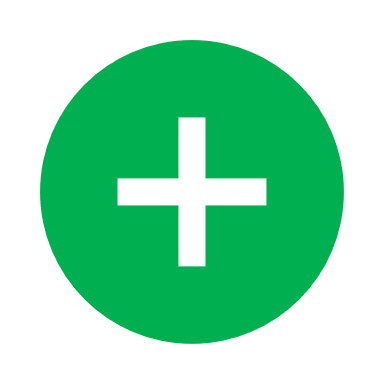 |
| Sandborn (OCTAVE) (Sandborn et al., 2017) | 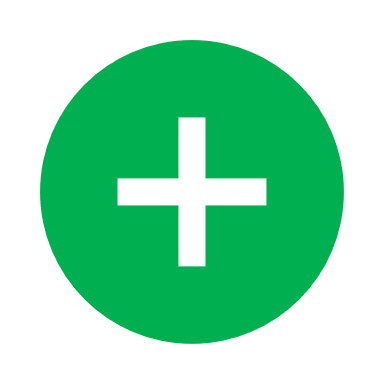 | 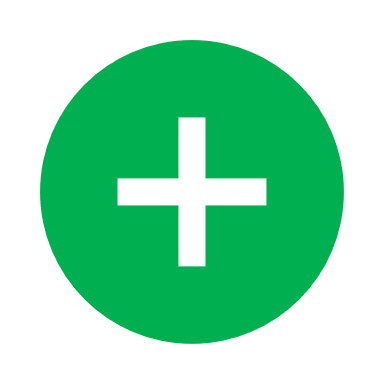 | 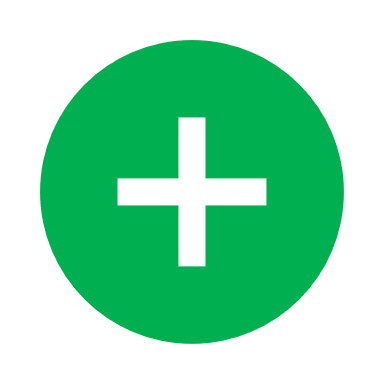 | 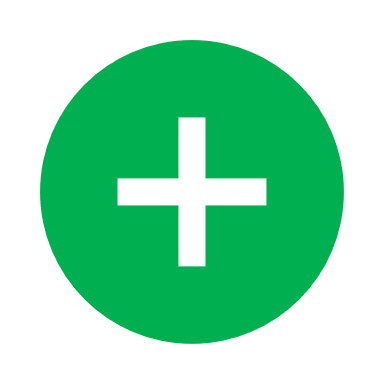 |
| Sandborn (OCTAVE) (Sandborn et al., 2017) | 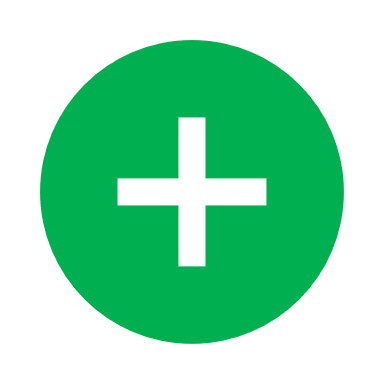 | 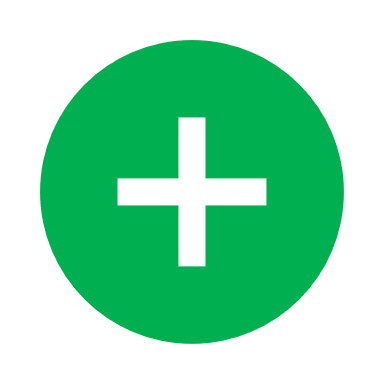 | 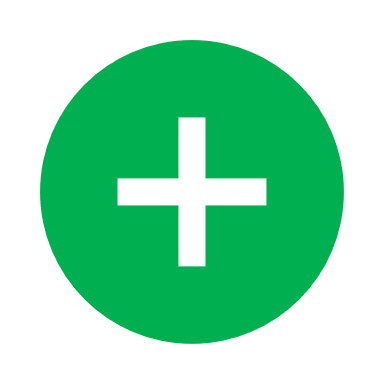 | 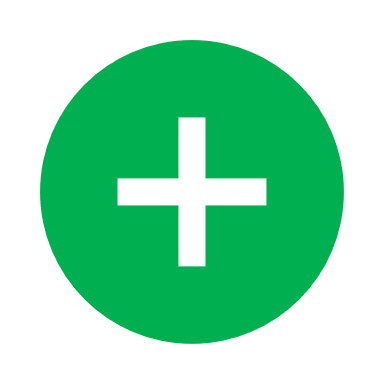 |
| Danese (UC1) (Danese et al., 2022) | 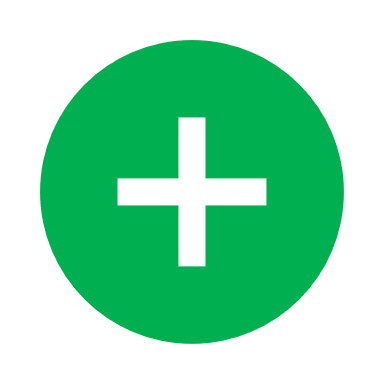 | 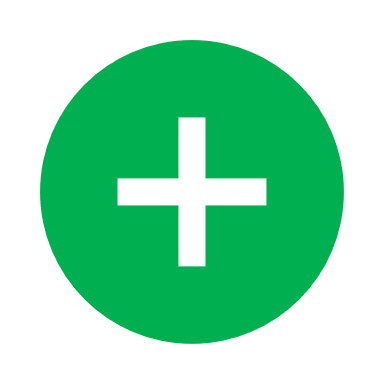 | 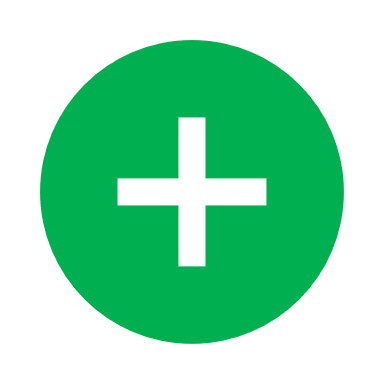 | 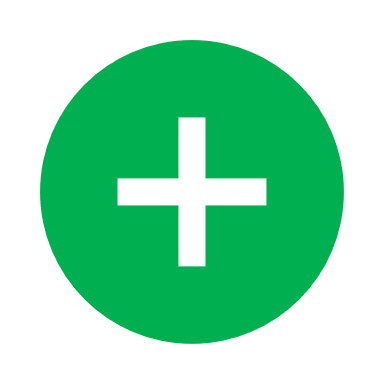 |
| Danese (UC2) (Danese et al., 2022) | 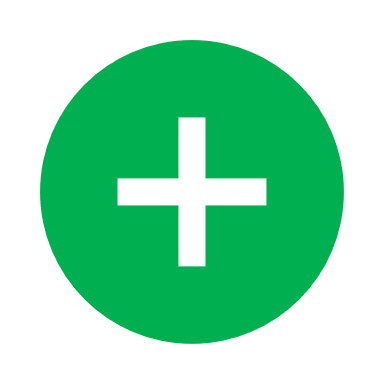 | 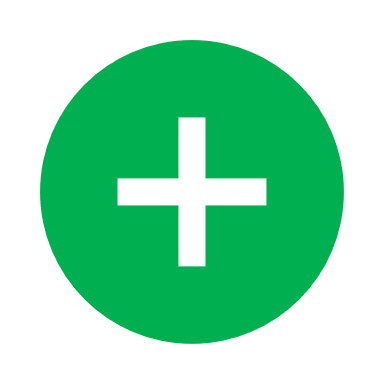 | 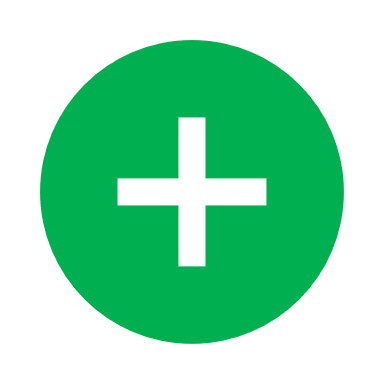 | 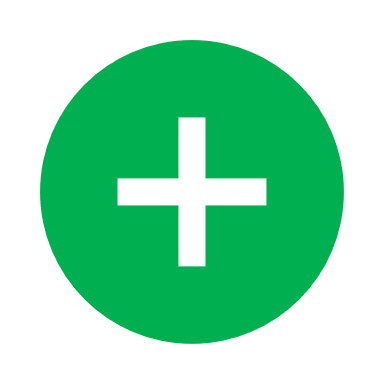 |
| Loftus (U-EXCEL) (Loftus et al., 2022) |  |  |  |  |
| Rheumatoid arthritis |  |  |  |  |
| Dougados (RA-BUILD) (Dougados et al., 2017) | 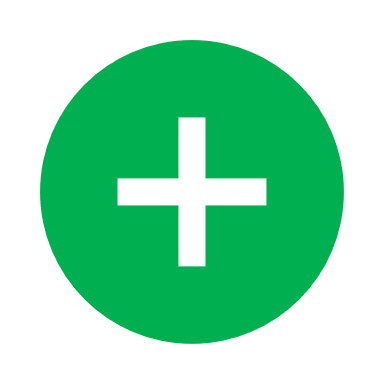 | 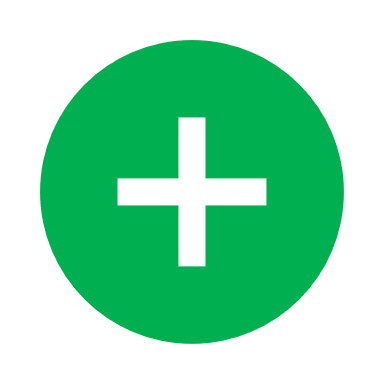 | 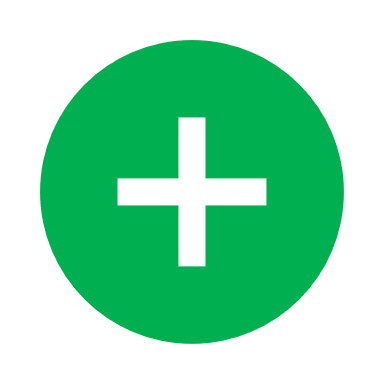 | 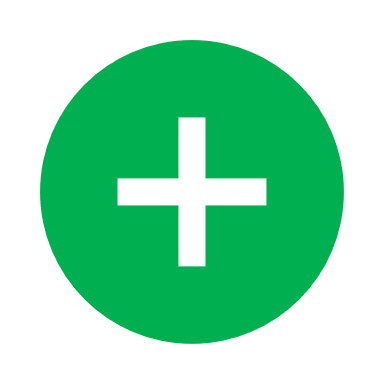 |
| Fleischmann (RA-BEGIN) (Fleischmann et al., 2017) | 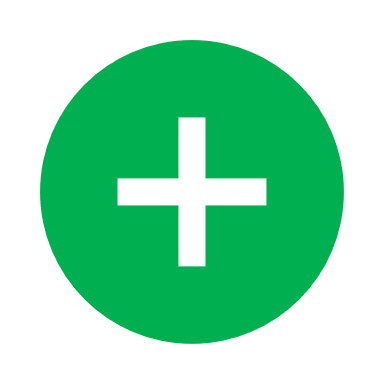 | 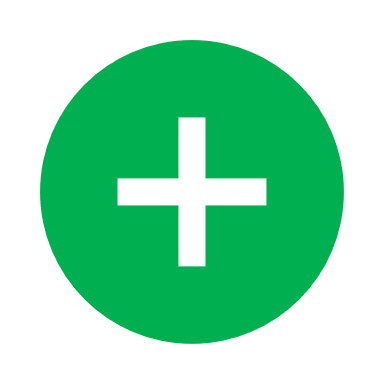 | 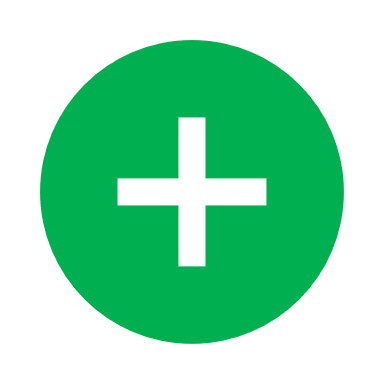 | 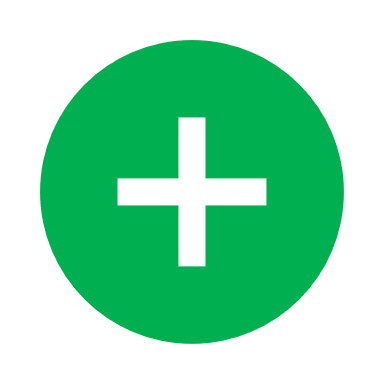 |
| Genovese (RA-BEACON) (Genovese et al., 2016a) | 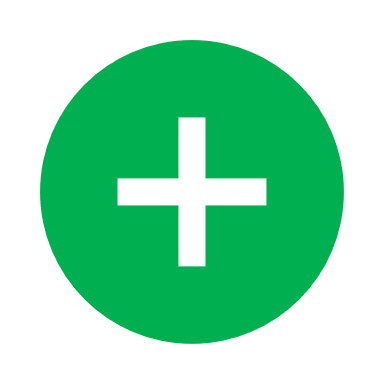 | 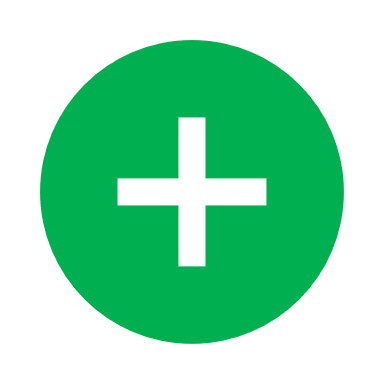 | 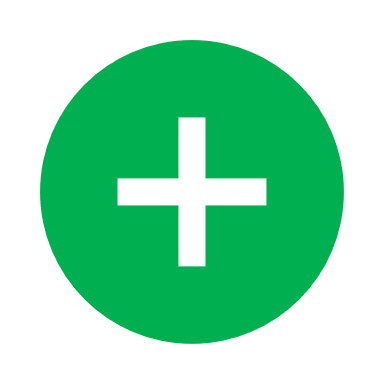 | 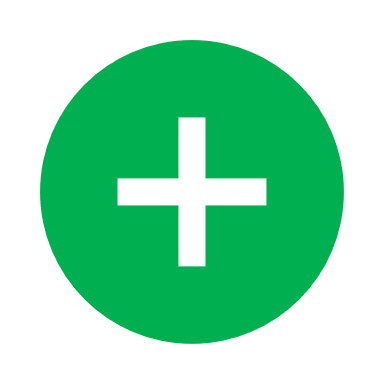 |
| Keystone (Keystone et al., 2015) | 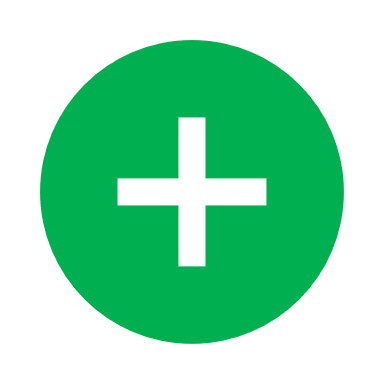 | 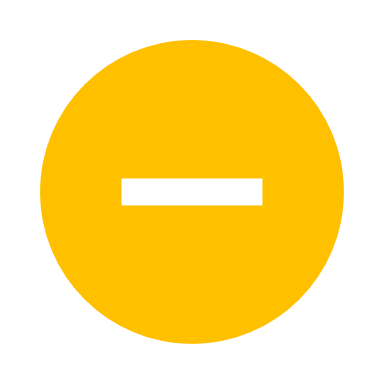 | 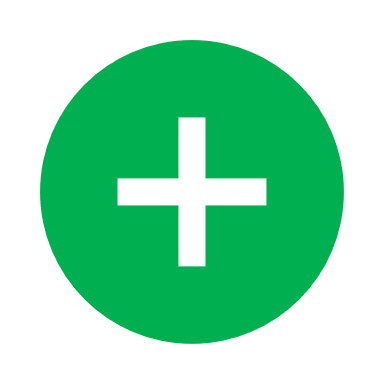 | 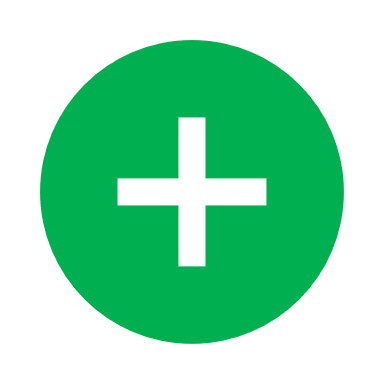 |
| Taylor (RA-BEAM) (Taylor et al., 2017) | 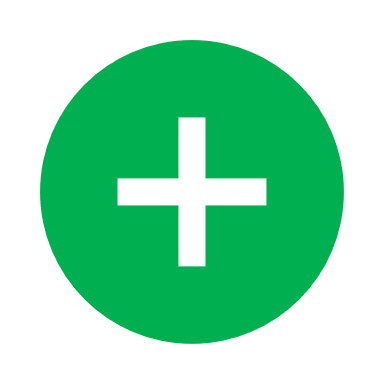 | 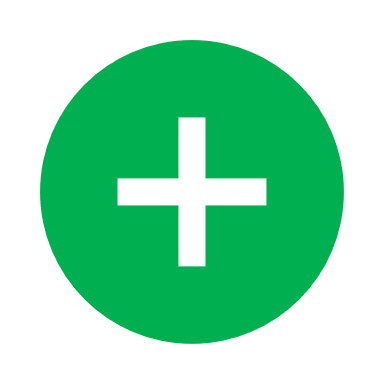 | 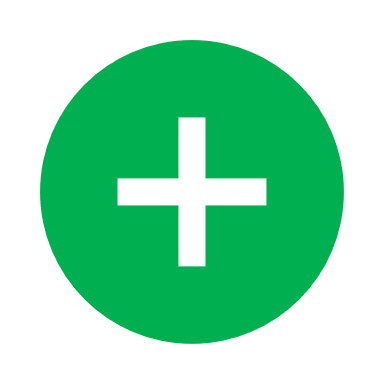 | 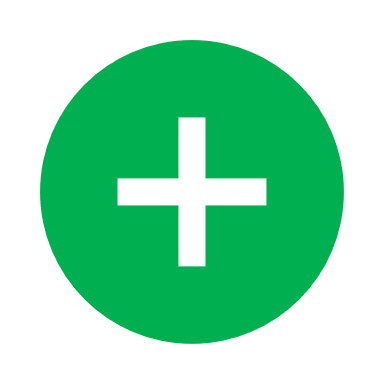 |
| Fleischmann and Damjanov (Fleischmann et al., 2015) | 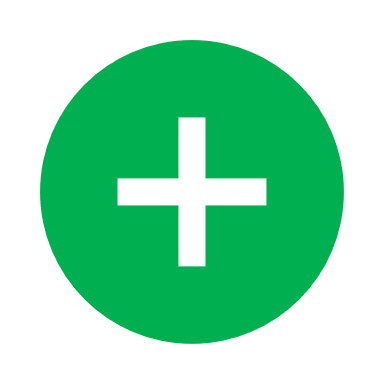 | 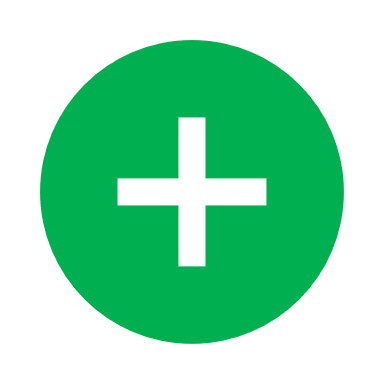 | 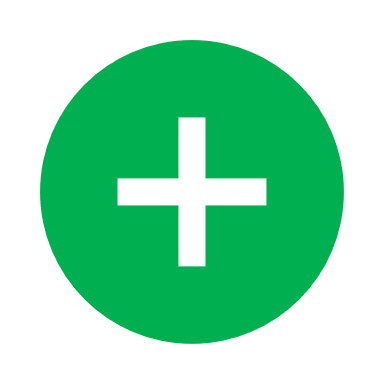 | 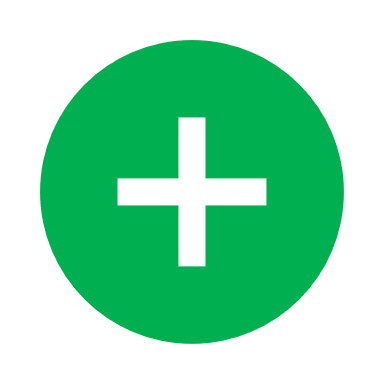 |
| Genovese and van Vollenhoven (Genovese et al., 2016b) | 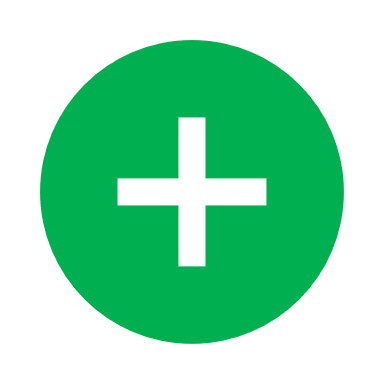 | 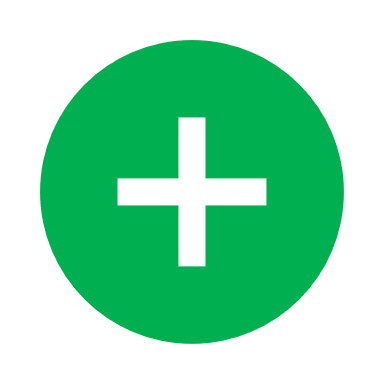 | 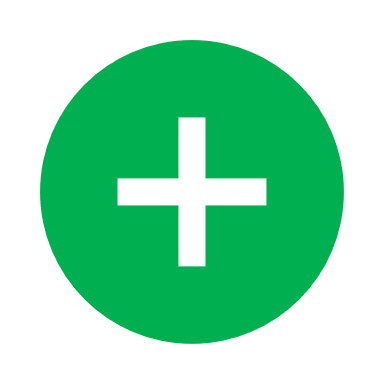 | 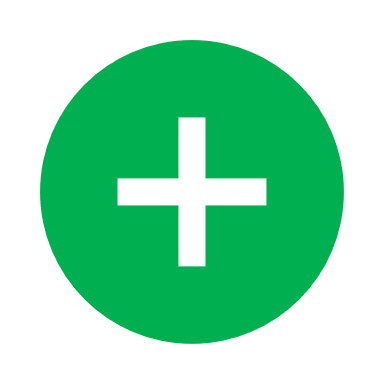 |
| Genovese (FINCH 2) (Genovese et al., 2019) | 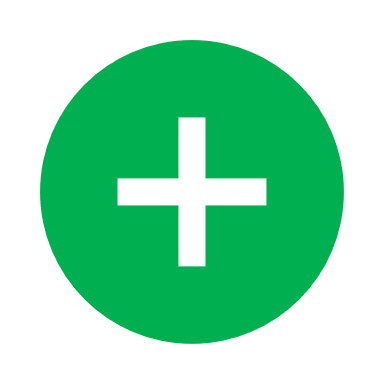 | 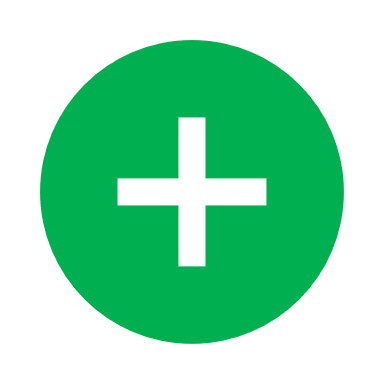 | 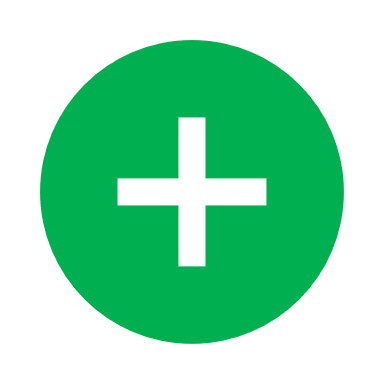 | 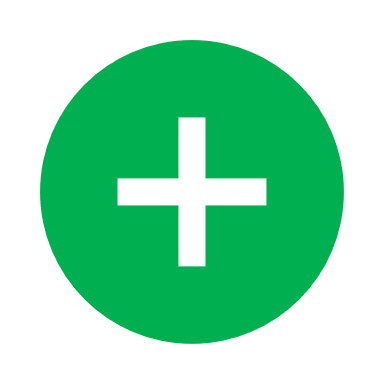 |
| Kavanaugh (DARWIN 2) (Kavanaugh et al., 2017) | 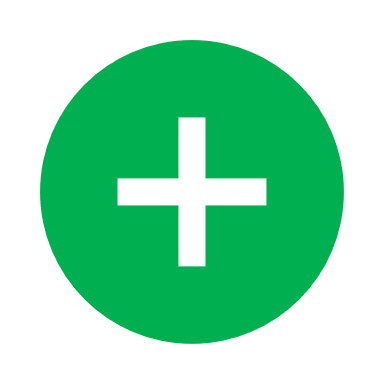 | 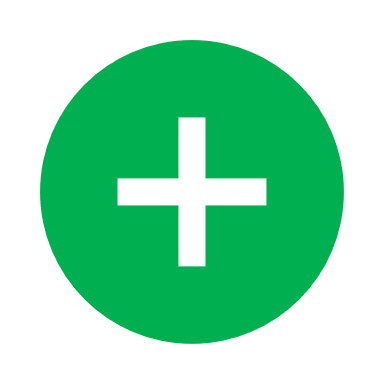 | 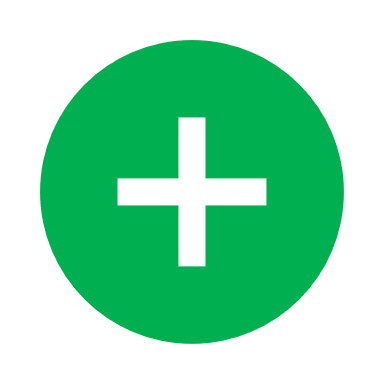 | 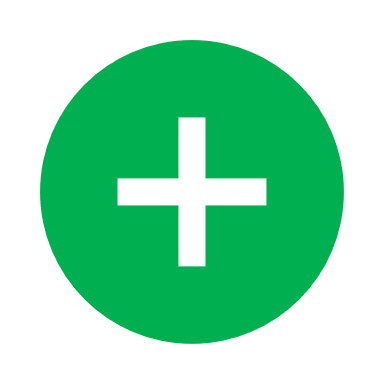 |
| Westhovens (DARWIN 1) (Westhovens et al., 2017) | 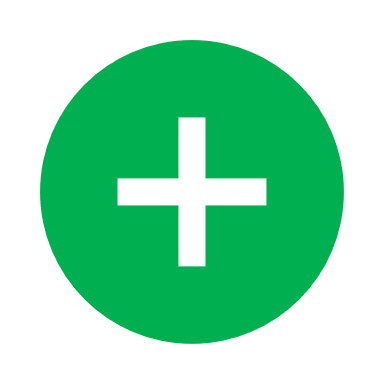 | 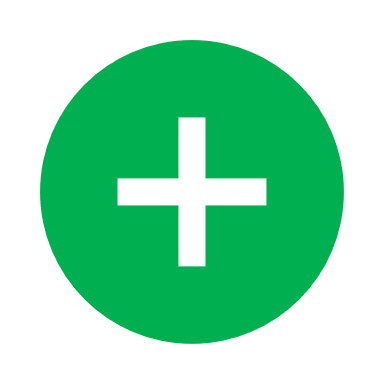 | 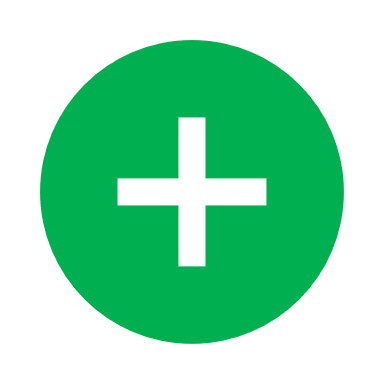 | 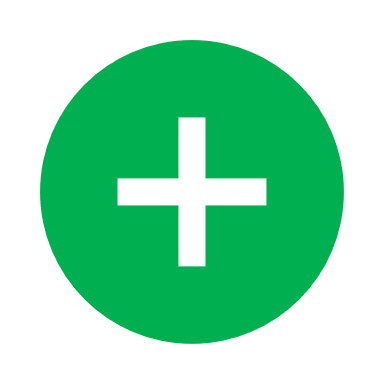 |
| Kivitz (Kivitz et al., 2017) | 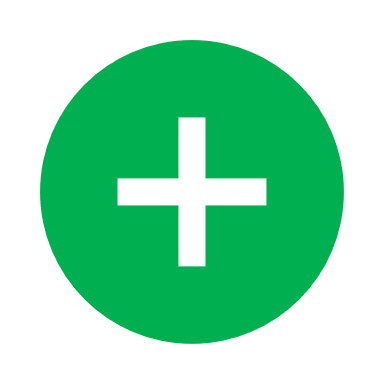 | 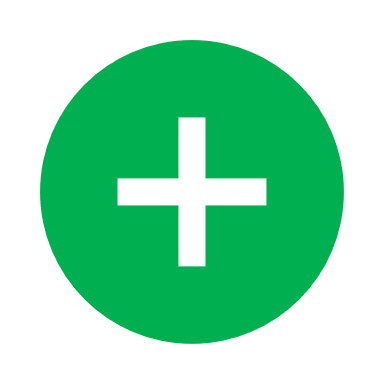 | 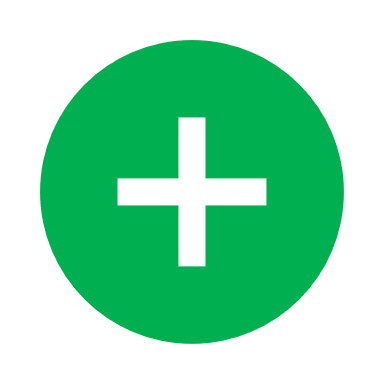 | 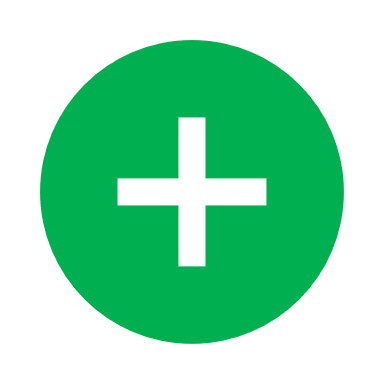 |
| Takeuchi (RAJ4) (Takeuchi et al., 2019) | 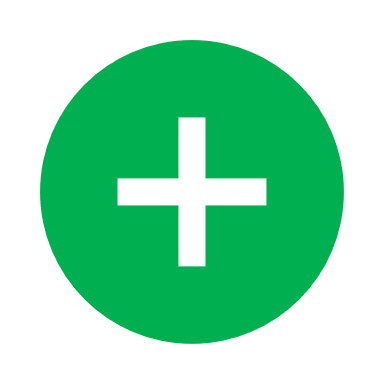 | 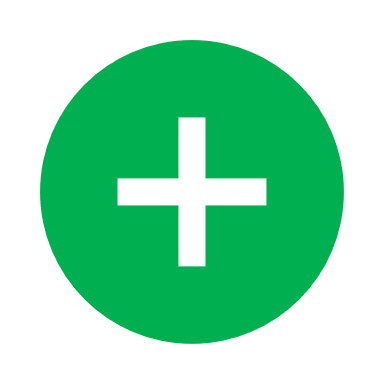 | 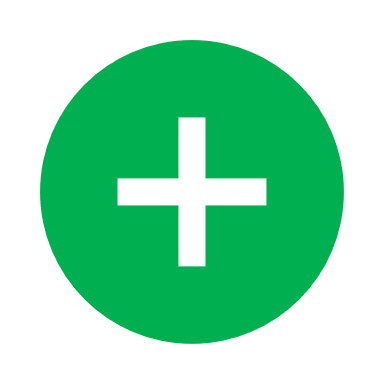 | 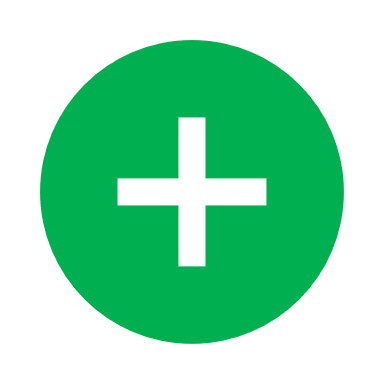 |
| Takeuchi and Tanaka (Takeuchi et al., 2016) | 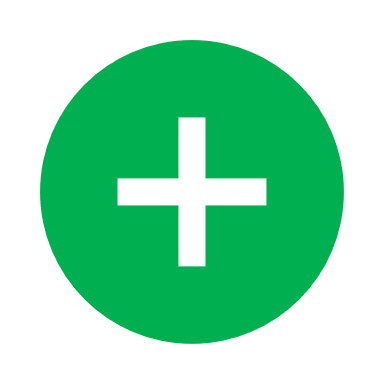 | 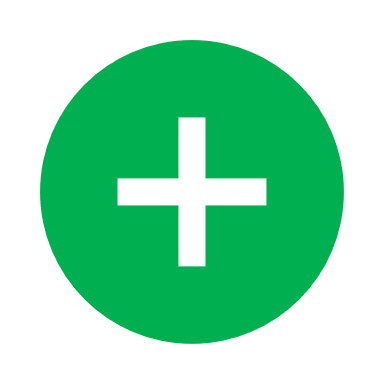 | 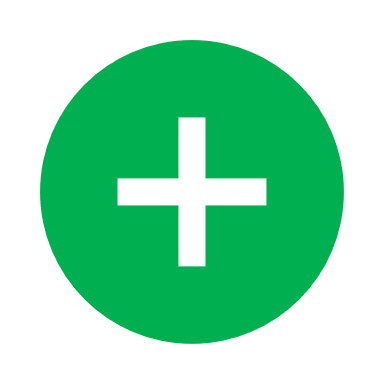 | 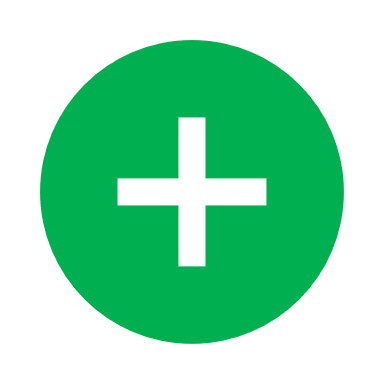 |
| Fleischmann (ORAL Solo) (Fleischmann et al., 2012) | 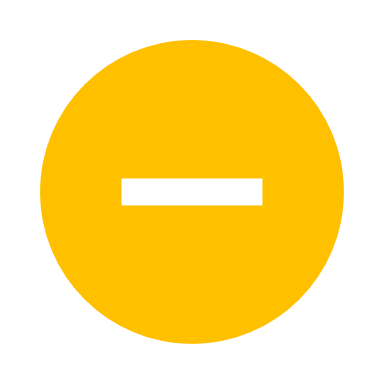 | 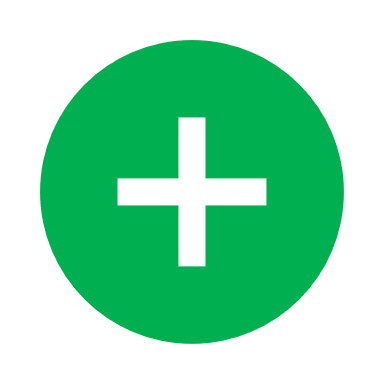 | 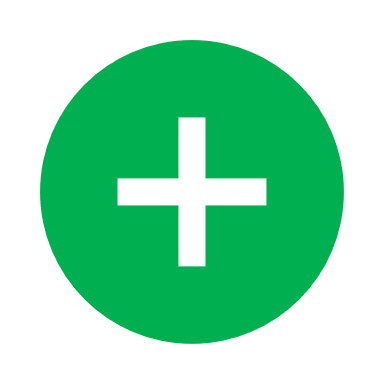 | 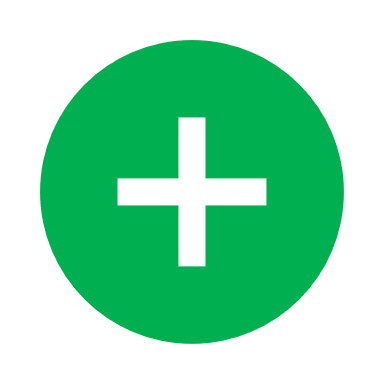 |
| Kremer (ORAL Sync) (Kremer et al., 2013) | 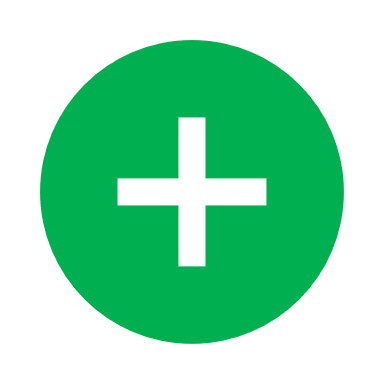 | 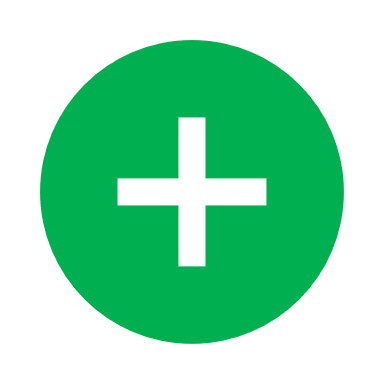 | 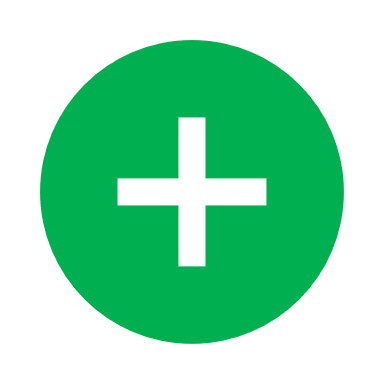 | 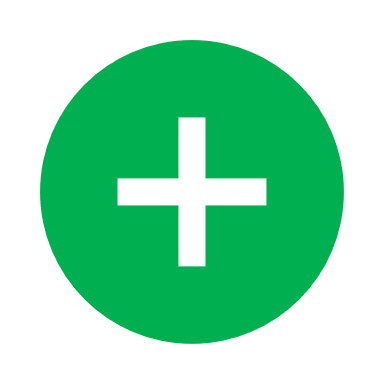 |
| Lee (Lee et al., 2014) | 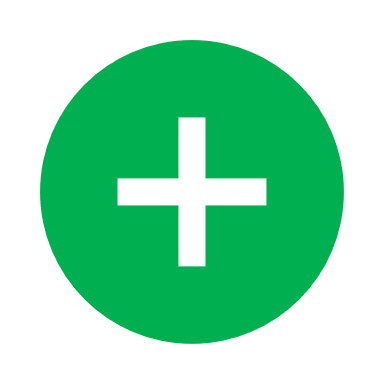 | 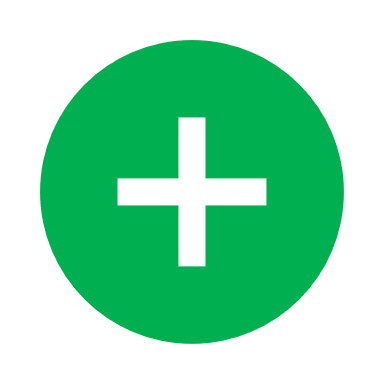 | 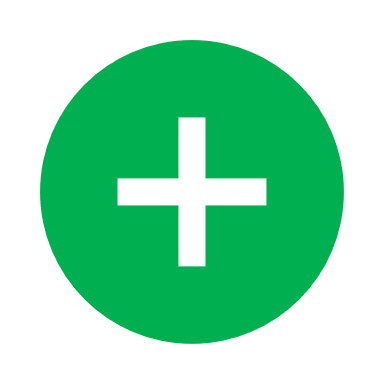 | 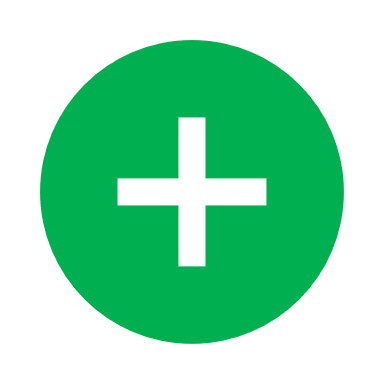 |
| Tanaka and Takeuchi (Tanaka et al., 2015) | 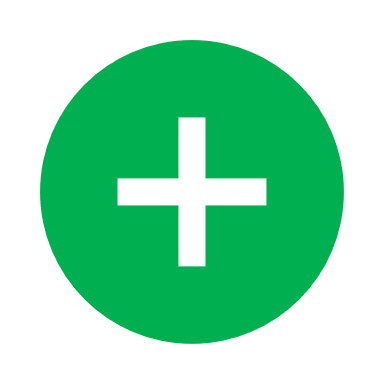 |  |  |  |
| van der Heijde (ORAL Scan) (van der Heijde et al., 2013) |  |  |  |  |
| van Vollenhoven (ORAL Standard) (van Vollenhoven et al., 2012) |  |  |  |  |
| Burmester (SELECT-NEXT) (Burmester et al., 2018) |  |  |  |  |
| Genovese (SELECT-BEYOND) (Genovese et al., 2018) |  |  |  |  |
| Smolen (SELECT-MONOTHERAPY) (Smolen et al., 2019) |  |  |  |  |
| Axial spondyloarthritis |  |  |  |  |
| Deodhar (Deodhar et al., 2021) |  |  |  |  |
| van der Heijde (van der Heijde et al., 2017) |  |  |  |  |
| van der Heijde (SELECT-AXIS 1) (van der Heijde et al., 2019) |  |  |  |  |
| van der Heijde (SELECT-AXIS 2) (AS) (van der Heijde et al., 2022) |  |  |  |  |
| Deodhar (SELECT-AXIS 2) (nr-axSpA) (Deodhar et al., 2022) |  |  |  |  |
| Psoriasis and psoriatic arthritis |  |  |  |  |
| Abe (Abe et al., 2017) |  |  |  |  |
| Bachelez (Bachelez et al., 2015) |  |  |  |  |
| Papp (OPT Pivotal 1) (Papp et al., 2015) |  |  |  |  |
| Papp (OPT Pivotal 2) (Papp et al., 2015) |  |  |  |  |
| Zhang (Zhang et al., 2017) |  |  |  |  |
| Mease (EQUATOR) (Mease et al., 2018) |  |  |  |  |
| Gladman (Gladman et al., 2017) |  |  |  |  |
| Leng (Leng et al., 2023) |  |  |  |  |
| Mease (OPAL Broaden) (Mease et al., 2017) |  |  |  |  |
| McInnes (SELECT-PsA 1) (McInnes et al., 2021) |  |  |  |  |
| Mease (SELECT-PsA 2) (Mease et al., 2021) |  |  |  |  |

### Evidence network diagram for inflammatory bowel disease

### Evidence network diagram of rheumatoid arthritis

### Evidence network diagram of axial spondyloarthritis

### Evidence network diagram of psoriasis and psoriatic arthritis

## Figure S1. Evidence network diagram of network meta-analysis comparisons

| Filgotinib 100mg QD | 2.21 (0.40, 12.34) | 0.30 (0.00, 30.76) | 0.85 (0.01, 61.60) | 0.83 (0.04, 16.05) | 3.32 (0.16, 67.58) | 0.88 (0.08, 10.33) |
| --- | --- | --- | --- | --- | --- | --- |
| 0.45 (0.08, 2.53) | Filgotinib 200mg QD | 0.13 (0.00, 12.93) | 0.38 (0.01, 25.73) | 0.38 (0.02, 6.46) | 1.50 (0.08, 27.25) | 0.40 (0.04, 4.05) |
| 3.37 (0.03, 349.17) | 7.44 (0.08, 715.88) | Ivarmacitinib | 2.85 (0.01, 557.01) | 2.79 (0.04, 199.51) | 11.17 (0.15, 827.17) | 2.98 (0.06, 152.34) |
| 1.18 (0.02, 86.11) | 2.61 (0.04, 175.45) | 0.35 (0.00, 68.58) | Tofacitinib 5mg BID | 0.98 (0.03, 32.88) | 3.92 (0.08, 197.94) | 1.04 (0.03, 35.03) |
| 1.21 (0.06, 23.35) | 2.66 (0.15, 45.83) | 0.36 (0.01, 25.57) | 1.02 (0.03, 34.22) | Tofacitinib 10mg BID | 4.00 (0.36, 44.19) | 1.07 (0.20, 5.56) |
| 0.30 (0.01, 6.14) | 0.67 (0.04, 12.09) | 0.09 (0.00, 6.62) | 0.26 (0.01, 12.87) | 0.25 (0.02, 2.76) | Upadacitinib 45mg | 0.27 (0.05, 1.52) |
| 1.13 (0.10, 13.25) | 2.50 (0.25, 25.34) | 0.34 (0.01, 17.20) | 0.96 (0.03, 32.12) | 0.94 (0.18, 4.90) | 3.76 (0.66, 21.46) | Placebo |

### A. League plot for inflammatory bowel disease

| Baricitinib  2mg QD | 1.42  (0.53,3.84) | 1.02  (0.09,11.31) | 0.41  (0.05,3.54) | 0.48  (0.06,3.66) | 2.49  (0.44,14.19) | 1.10  (0.18,6.78) | 0.81  (0.17,3.94) | 0.85  (0.18,4.11) | 0.67  (0.10,4.46) | 1.59  (0.27,9.39) | 0.41  (0.12,1.40) |
| --- | --- | --- | --- | --- | --- | --- | --- | --- | --- | --- | --- |
| 0.70  (0.26,1.90) | Baricitinib  4mg QD | 0.71  (0.07,6.91) | 0.29  (0.04,2.13) | 0.34  (0.05,2.18) | 1.75  (0.38,8.17) | 0.77  (0.15,3.94) | 0.57  (0.15,2.22) | 0.60  (0.16,2.31) | 0.47  (0.08,2.62) | 1.12  (0.23,5.43) | 0.29  (0.12,0.73) |
| 0.98  (0.09,10.94) | 1.40  (0.14,13.54) | Decernotinib | 0.40  (0.03,6.18) | 0.47  (0.03,6.57) | 2.45  (0.22,27.44) | 1.08  (0.09,12.82) | 0.80  (0.08,8.00) | 0.84  (0.08,8.35) | 0.65  (0.05,8.26) | 1.57  (0.14,17.98) | 0.41  (0.05,3.23) |
| 2.45  (0.28,21.31) | 3.49  (0.47,25.92) | 2.49  (0.16,38.41) | Filgotinib  100mg QD | 1.17  (0.28,4.90) | 6.12  (0.70,53.46) | 2.70  (0.29,25.14) | 2.00  (0.26,15.37) | 2.09  (0.27,16.04) | 1.63  (0.16,16.31) | 3.90  (0.43,35.13) | 1.01  (0.17,6.00) |
| 2.09  (0.27,16.06) | 2.98  (0.46,19.34) | 2.13  (0.15,29.76) | 0.85  (0.20,3.58) | Filgotinib  200mg QD | 5.22  (0.68,40.31) | 2.31  (0.28,19.03) | 1.70  (0.25,11.49) | 1.79  (0.27,11.99) | 1.39  (0.16,12.39) | 3.33  (0.42,26.54) | 0.86  (0.17,4.39) |
| 0.40  (0.07,2.28) | 0.57  (0.12,2.66) | 0.41  (0.04,4.56) | 0.16  (0.02,1.43) | 0.19  (0.02,1.48) | Peficitinib  100mg QD | 0.44  (0.19,1.04) | 0.33  (0.07,1.59) | 0.34  (0.07,1.66) | 0.27  (0.04,1.80) | 0.64  (0.11,3.79) | 0.17  (0.05,0.57) |
| 0.91  (0.15,5.59) | 1.29  (0.25,6.58) | 0.92  (0.08,10.93) | 0.37  (0.04,3.45) | 0.43  (0.05,3.58) | 2.27  (0.96,5.33) | Peficitinib  150mg QD | 0.74  (0.14,3.93) | 0.77  (0.15,4.10) | 0.60  (0.08,4.38) | 1.45  (0.23,9.28) | 0.37  (0.10,1.43) |
| 1.23  (0.25,5.95) | 1.75  (0.45,6.77) | 1.25  (0.13,12.47) | 0.50  (0.07,3.86) | 0.59  (0.09,3.95) | 3.06  (0.63,14.96) | 1.35  (0.25,7.19) | Tofacitinib  5mg BID | 1.05  (0.58,1.88) | 0.82  (0.14,4.77) | 1.96  (0.38,9.93) | 0.51  (0.19,1.37) |
| 1.17  (0.24,5.65) | 1.67  (0.43,6.43) | 1.19  (0.12,11.86) | 0.48  (0.06,3.67) | 0.56  (0.08,3.76) | 2.92  (0.60,14.21) | 1.29  (0.24,6.83) | 0.95  (0.53,1.71) | Tofacitinib  10mg BID | 0.78  (0.13,4.53) | 1.87  (0.37,9.44) | 0.48  (0.18,1.30) |
| 1.50  (0.22,10.07) | 2.14  (0.38,11.97) | 1.53  (0.12,19.26) | 0.61  (0.06,6.12) | 0.72  (0.08,6.37) | 3.75  (0.56,25.28) | 1.65  (0.23,11.99) | 1.22  (0.21,7.13) | 1.28  (0.22,7.44) | Upadacitinib  15mg QD | 2.39  (0.83,6.89) | 0.62  (0.14,2.65) |
| 0.63  (0.11,3.71) | 0.89  (0.18,4.34) | 0.64  (0.06,7.33) | 0.26  (0.03,2.31) | 0.30  (0.04,2.39) | 1.57  (0.26,9.31) | 0.69  (0.11,4.44) | 0.51  (0.10,2.60) | 0.54  (0.11,2.71) | 0.42  (0.15,1.21) | Upadacitinib  30mg QD | 0.26  (0.07,0.94) |
| 2.43  (0.71,8.26) | 3.46  (1.38,8.67) | 2.47  (0.31,19.66) | 0.99  (0.17,5.89) | 1.16  (0.23,5.91) | 6.06  (1.76,20.82) | 2.67  (0.70,10.24) | 1.98  (0.73,5.34) | 2.07  (0.77,5.56) | 1.62  (0.38,6.94) | 3.87  (1.07,13.98) | Placebo |

### B. League plot for rheumatoid arthritis

| Tofacitinib 5mg BID | 2.98 (0.16, 54.57) | 2.33 (0.09, 60.84) | 0.99 (0.06, 15.96) |
| --- | --- | --- | --- |
| 0.34 (0.02, 6.13) | Tofacitinib 10mg BID | 0.78 (0.03, 22.71) | 0.33 (0.02, 6.06) |
| 0.43 (0.02, 11.17) | 1.28 (0.04, 37.13) | Upadacitinib 15mg QD | 0.42 (0.08, 2.33) |
| 1.01 (0.06, 16.32) | 3.02 (0.16, 55.19) | 2.36 (0.43, 12.95) | Placebo |

### C. League plot for axial spondyloarthritis

| Tofacitinib 5mg BID | 1.18 (0.57, 2.46) | 1.40 (0.05, 42.62) | 0.63 (0.12, 3.16) | 1.17 (0.25, 5.49) | 0.45 (0.15, 1.41) |
| --- | --- | --- | --- | --- | --- |
| 0.85 (0.41, 1.77) | Tofacitinib 10mg BID | 1.19 (0.04, 36.31) | 0.53 (0.10, 2.70) | 0.99 (0.21, 4.70) | 0.38 (0.12, 1.21) |
| 0.71 (0.02, 21.66) | 0.84 (0.03, 25.61) | Filgotinib200mg QD | 0.45 (0.01, 13.62) | 0.83 (0.03, 24.57) | 0.32 (0.01, 8.08) |
| 1.60 (0.32, 8.07) | 1.88 (0.37, 9.59) | 2.24 (0.07, 68.48) | Upadacitinib 15mg QD | 1.86 (0.73, 4.76) | 0.72 (0.23, 2.30) |
| 0.86 (0.18, 4.04) | 1.01 (0.21, 4.79) | 1.20 (0.04, 35.56) | 0.54 (0.21, 1.37) | Upadacitinib 30mg QD | 0.39 (0.14, 1.11) |
| 2.20 (0.71, 6.87) | 2.60 (0.82, 8.19) | 3.09 (0.12, 77.32) | 1.38 (0.44, 4.37) | 2.57 (0.90, 7.36) | Placebo |

### D. League plot for psoriasis and psoriatic arthritis

## Figure S2. League plots of the incidence of Herpes zoster infection for all drugs comparing

### Cumulative rank probability plot of immune-mediated inflammatory disease

### Cumulative rank probability plot of inflammatory bowel disease

### Cumulative rank probability plot of rheumatoid arthritis

### Cumulative rank probability plot of axial spondyloarthritis

### Cumulative rank probability plot of psoriasis and psoriatic arthritis

## Figure S3. Cumulative rank probability plots

### SUCRA Ranking plot of inflammatory bowel disease

### SUCRA Ranking plot rheumatoid arthritis

### SUCRA Ranking plot axial spondyloarthritis

### SUCRA Ranking plot psoriasis and psoriatic arthritis

## Figure S4. Cluster SUCRA ranking plot. SUCRA, Surface under cumulative ranking.

### Forest plot for immune-mediated inflammatory disease

### Forest plot for inflammatory bowel disease

### Forest plot for Rheumatoid arthritis

### Forest plot for axial spondyloarthritis

### Forest plot for psoriasis and psoriatic arthritis

## Figure S5. Forest plot with Predictive interval plot for all outcomes

In these plots, the diamonds represent the mean of summary odds ratios (ORs) for each comparison; the black lines represent the 95% confidence intervals of ORs (95%CI); the red lines indicate respective 95% Predictive Intervals (PrI), which provide an interval within which the estimate of a future study is expected to be. The blue vertical line is the line of no effect (OR equal to 1).

## Table S3. Evaluation of inconsistency.

| **Network outcome** | **Chi-square** | **P value** |
| --- | --- | --- |
| **Immune-mediated inflammatory disease** | 2.16 | 0.8267 |
| **Inflammatory bowel disease** | 2.21 | 0.1368 |
| **Rheumatoid arthritis** | 0.43 | 0.8078 |
| **Axial spondyloarthritis** | 0.00 | 0.9938 |
| **Psoriasis and psoriatic arthritis** | 0.59 | 0.4407 |

### Publication bias of immune-mediated inflammatory disease.

A~N indicating: Baricitinib2mgQD, Baricitinib4mgQD, Decernotinib, Filgotinib100mgQD, Filgotinib200mgQD, Ivarmacitinib, Peficitinib100mgQD, Peficitinib150mgQD, Tofacitinib5mgBID, Tofacitinib10mgBID, Upadacitinib15mgQD, Upadacitinib30mgQD, Upadacitinib45mgQD, Placebo.

### Publication bias of inflammatory bowel disease.

A~G indicating: Filgotinib100mgQD, Filgotinib200mgQD, Ivarmacitinib, Tofacitinib5mgBID, Tofacitinib10mgBID, Upadacitinib45mgQD, Placebo.

### Publication bias of rheumatoid arthritis

A~L indicating: Baricitinib2mgQD, Baricitinib4mgQD, Decernotinib, Filgotinib100mgQD, Filgotinib200mgQD, Peficitinib100mgQD, Peficitinib150mgQD, Tofacitinib5mgBID, Tofacitinib10mgBID, Upadacitinib15mgQD, Upadacitinib30mgQD, Placebo.

### Publication bias of axial spondyloarthritis

A~D indicating: Tofacitinib5mgBID, Tofacitinib10mgBID, Upadacitinib15mgQD, Placebo.

### Publication bias of psoriasis and psoriatic arthritis

A~F indicating: Tofacitinib5mgBID, Tofacitinib10mgBID, Filgotinib200mgQD, Upadacitinib15mgQD, Upadacitinib30mgQD, Placebo.

## Figure S6. Publication bias

**REFERENCES:**

Abe, M., Nishigori, C., Torii, H., Ihn, H., Ito, K., Nagaoka, M., Isogawa, N., Kawaguchi, I., Tomochika, Y., Kobayashi, M., Tallman, A.M., and Papp, K.A. (2017). Tofacitinib for the treatment of moderate to severe chronic plaque psoriasis in Japanese patients: Subgroup analyses from a randomized, placebo-controlled phase 3 trial. *J Dermatol* 44**,** 1228-1237.

Bachelez, H., Van De Kerkhof, P.C., Strohal, R., Kubanov, A., Valenzuela, F., Lee, J.H., Yakusevich, V., Chimenti, S., Papacharalambous, J., Proulx, J., Gupta, P., Tan, H., Tawadrous, M., Valdez, H., and Wolk, R. (2015). Tofacitinib versus etanercept or placebo in moderate-to-severe chronic plaque psoriasis: a phase 3 randomised non-inferiority trial. *Lancet* 386**,** 552-561.

Burmester, G.R., Kremer, J.M., Van Den Bosch, F., Kivitz, A., Bessette, L., Li, Y., Zhou, Y., Othman, A.A., Pangan, A.L., and Camp, H.S. (2018). Safety and efficacy of upadacitinib in patients with rheumatoid arthritis and inadequate response to conventional synthetic disease-modifying anti-rheumatic drugs (SELECT-NEXT): a randomised, double-blind, placebo-controlled phase 3 trial. *Lancet* 391**,** 2503-2512.

Chen, B., Zhong, J., Li, X., Pan, F., Ding, Y., Zhang, Y., Chen, H., Liu, F., Zhang, Z., Zhang, L., Drozda, R., Oliinyk, O., Goh, A.H., Chen, X., Sun, X., Rubin, D.T., Sandborn, W.J., and Chen, M. (2022). Efficacy and Safety of Ivarmacitinib in Patients With Moderate-to-Severe, Active, Ulcerative Colitis: A Phase II Study. *Gastroenterology* 163**,** 1555-1568.

Danese, S., Vermeire, S., Zhou, W., Pangan, A.L., Siffledeen, J., Greenbloom, S., Hébuterne, X., D'haens, G., Nakase, H., Panés, J., Higgins, P.D.R., Juillerat, P., Lindsay, J.O., Loftus, E.V., Jr., Sandborn, W.J., Reinisch, W., Chen, M.H., Sanchez Gonzalez, Y., Huang, B., Xie, W., Liu, J., Weinreich, M.A., and Panaccione, R. (2022). Upadacitinib as induction and maintenance therapy for moderately to severely active ulcerative colitis: results from three phase 3, multicentre, double-blind, randomised trials. *Lancet* 399**,** 2113-2128.

Deodhar, A., Sliwinska-Stanczyk, P., Xu, H., Baraliakos, X., Gensler, L.S., Fleishaker, D., Wang, L., Wu, J., Menon, S., Wang, C., Dina, O., Fallon, L., Kanik, K.S., and Van Der Heijde, D. (2021). Tofacitinib for the treatment of ankylosing spondylitis: a phase III, randomised, double-blind, placebo-controlled study. *Ann Rheum Dis* 80**,** 1004-1013.

Deodhar, A., Van Den Bosch, F., Poddubnyy, D., Maksymowych, W.P., Van Der Heijde, D., Kim, T.H., Kishimoto, M., Blanco, R., Duan, Y., Li, Y., Pangan, A.L., Wung, P., and Song, I.H. (2022). Upadacitinib for the treatment of active non-radiographic axial spondyloarthritis (SELECT-AXIS 2): a randomised, double-blind, placebo-controlled, phase 3 trial. *Lancet* 400**,** 369-379.

Dougados, M., Van Der Heijde, D., Chen, Y.C., Greenwald, M., Drescher, E., Liu, J., Beattie, S., Witt, S., De La Torre, I., Gaich, C., Rooney, T., Schlichting, D., De Bono, S., and Emery, P. (2017). Baricitinib in patients with inadequate response or intolerance to conventional synthetic DMARDs: results from the RA-BUILD study. *Ann Rheum Dis* 76**,** 88-95.

Feagan, B.G., Danese, S., Loftus, E.V., Jr., Vermeire, S., Schreiber, S., Ritter, T., Fogel, R., Mehta, R., Nijhawan, S., Kempiński, R., Filip, R., Hospodarskyy, I., Seidler, U., Seibold, F., Beales, I.L.P., Kim, H.J., Mcnally, J., Yun, C., Zhao, S., Liu, X., Hsueh, C.H., Tasset, C., Besuyen, R., Watanabe, M., Sandborn, W.J., Rogler, G., Hibi, T., and Peyrin-Biroulet, L. (2021). Filgotinib as induction and maintenance therapy for ulcerative colitis (SELECTION): a phase 2b/3 double-blind, randomised, placebo-controlled trial. *Lancet* 397**,** 2372-2384.

Fleischmann, R., Kremer, J., Cush, J., Schulze-Koops, H., Connell, C.A., Bradley, J.D., Gruben, D., Wallenstein, G.V., Zwillich, S.H., and Kanik, K.S. (2012). Placebo-controlled trial of tofacitinib monotherapy in rheumatoid arthritis. *N Engl J Med* 367**,** 495-507.

Fleischmann, R., Schiff, M., Van Der Heijde, D., Ramos-Remus, C., Spindler, A., Stanislav, M., Zerbini, C.A., Gurbuz, S., Dickson, C., De Bono, S., Schlichting, D., Beattie, S., Kuo, W.L., Rooney, T., Macias, W., and Takeuchi, T. (2017). Baricitinib, Methotrexate, or Combination in Patients With Rheumatoid Arthritis and No or Limited Prior Disease-Modifying Antirheumatic Drug Treatment. *Arthritis Rheumatol* 69**,** 506-517.

Fleischmann, R.M., Damjanov, N.S., Kivitz, A.J., Legedza, A., Hoock, T., and Kinnman, N. (2015). A randomized, double-blind, placebo-controlled, twelve-week, dose-ranging study of decernotinib, an oral selective JAK-3 inhibitor, as monotherapy in patients with active rheumatoid arthritis. *Arthritis Rheumatol* 67**,** 334-343.

Genovese, M.C., Fleischmann, R., Combe, B., Hall, S., Rubbert-Roth, A., Zhang, Y., Zhou, Y., Mohamed, M.F., Meerwein, S., and Pangan, A.L. (2018). Safety and efficacy of upadacitinib in patients with active rheumatoid arthritis refractory to biologic disease-modifying anti-rheumatic drugs (SELECT-BEYOND): a double-blind, randomised controlled phase 3 trial. *Lancet* 391**,** 2513-2524.

Genovese, M.C., Kalunian, K., Gottenberg, J.E., Mozaffarian, N., Bartok, B., Matzkies, F., Gao, J., Guo, Y., Tasset, C., Sundy, J.S., De Vlam, K., Walker, D., and Takeuchi, T. (2019). Effect of Filgotinib vs Placebo on Clinical Response in Patients With Moderate to Severe Rheumatoid Arthritis Refractory to Disease-Modifying Antirheumatic Drug Therapy: The FINCH 2 Randomized Clinical Trial. *Jama* 322**,** 315-325.

Genovese, M.C., Kremer, J., Zamani, O., Ludivico, C., Krogulec, M., Xie, L., Beattie, S.D., Koch, A.E., Cardillo, T.E., Rooney, T.P., Macias, W.L., De Bono, S., Schlichting, D.E., and Smolen, J.S. (2016a). Baricitinib in Patients with Refractory Rheumatoid Arthritis. *N Engl J Med* 374**,** 1243-1252.

Genovese, M.C., Van Vollenhoven, R.F., Pacheco-Tena, C., Zhang, Y., and Kinnman, N. (2016b). VX-509 (Decernotinib), an Oral Selective JAK-3 Inhibitor, in Combination With Methotrexate in Patients With Rheumatoid Arthritis. *Arthritis Rheumatol* 68**,** 46-55.

Gladman, D., Rigby, W., Azevedo, V.F., Behrens, F., Blanco, R., Kaszuba, A., Kudlacz, E., Wang, C., Menon, S., Hendrikx, T., and Kanik, K.S. (2017). Tofacitinib for Psoriatic Arthritis in Patients with an Inadequate Response to TNF Inhibitors. *New England Journal of Medicine* 377**,** 1525-1536.

Kavanaugh, A., Kremer, J., Ponce, L., Cseuz, R., Reshetko, O.V., Stanislavchuk, M., Greenwald, M., Van Der Aa, A., Vanhoutte, F., Tasset, C., and Harrison, P. (2017). Filgotinib (GLPG0634/GS-6034), an oral selective JAK1 inhibitor, is effective as monotherapy in patients with active rheumatoid arthritis: results from a randomised, dose-finding study (DARWIN 2). *Ann Rheum Dis* 76**,** 1009-1019.

Keystone, E.C., Taylor, P.C., Drescher, E., Schlichting, D.E., Beattie, S.D., Berclaz, P.Y., Lee, C.H., Fidelus-Gort, R.K., Luchi, M.E., Rooney, T.P., Macias, W.L., and Genovese, M.C. (2015). Safety and efficacy of baricitinib at 24 weeks in patients with rheumatoid arthritis who have had an inadequate response to methotrexate. *Ann Rheum Dis* 74**,** 333-340.

Kivitz, A.J., Gutierrez-Urena, S.R., Poiley, J., Genovese, M.C., Kristy, R., Shay, K., Wang, X., Garg, J.P., and Zubrzycka-Sienkiewicz, A. (2017). Peficitinib, a JAK Inhibitor, in the Treatment of Moderate-to-Severe Rheumatoid Arthritis in Patients With an Inadequate Response to Methotrexate. *Arthritis Rheumatol* 69**,** 709-719.

Kremer, J., Li, Z.G., Hall, S., Fleischmann, R., Genovese, M., Martin-Mola, E., Isaacs, J.D., Gruben, D., Wallenstein, G., Krishnaswami, S., Zwillich, S.H., Koncz, T., Riese, R., and Bradley, J. (2013). Tofacitinib in combination with nonbiologic disease-modifying antirheumatic drugs in patients with active rheumatoid arthritis: a randomized trial. *Ann Intern Med* 159**,** 253-261.

Lee, E.B., Fleischmann, R., Hall, S., Wilkinson, B., Bradley, J.D., Gruben, D., Koncz, T., Krishnaswami, S., Wallenstein, G.V., Zang, C., Zwillich, S.H., and Van Vollenhoven, R.F. (2014). Tofacitinib versus methotrexate in rheumatoid arthritis. *N Engl J Med* 370**,** 2377-2386.

Leng, X., Lin, W., Liu, S., Kanik, K., Wang, C., Wan, W., Jiang, Z., Liu, Y., Liu, S., Zhang, Z., Zhang, Z., Xu, J., Tan, W., Hu, J., Li, J., Liu, J., Gunay, L.M., Dina, O., Kinch, C., and Zeng, X. (2023). Efficacy and safety of tofacitinib in Chinese patients with active psoriatic arthritis: a phase 3, randomised, double-blind, placebo-controlled study. *RMD Open* 9.

Loftus, E., Colombel, J.F., Lacerda, A.P., Peyrin-Biroulet, L., D'haens, G., Panaccione, R., Reinisch, W., Louis, E., Chen, M., Nakase, H., Greenbloom, S., Duvall, A., Sanchez Gonzalez, Y., Mohammed, M.-E.F., Rhee, S., Feng, T., Dubcenco, E., and Panes, J. (2022). Efficacy and safety of upadactinib induction therapy in patients with moderately to severely active Crohn's disease: Results from a randomized phase 3 U-EXCEL study. *United European Gastroenterol J* 10 (supplement 8)**,** 103-104.

Mcinnes, I.B., Anderson, J.K., Magrey, M., Merola, J.F., Liu, Y., Kishimoto, M., Jeka, S., Pacheco-Tena, C., Wang, X., Chen, L., Zueger, P., Liu, J., Pangan, A.L., and Behrens, F. (2021). Trial of Upadacitinib and Adalimumab for Psoriatic Arthritis. *N Engl J Med* 384**,** 1227-1239.

Mease, P., Coates, L.C., Helliwell, P.S., Stanislavchuk, M., Rychlewska-Hanczewska, A., Dudek, A., Abi-Saab, W., Tasset, C., Meuleners, L., Harrison, P., Besuyen, R., Van Der Aa, A., Mozaffarian, N., Greer, J.M., Kunder, R., Van Den Bosch, F., and Gladman, D.D. (2018). Efficacy and safety of filgotinib, a selective Janus kinase 1 inhibitor, in patients with active psoriatic arthritis (EQUATOR): results from a randomised, placebo-controlled, phase 2 trial. *Lancet* 392**,** 2367-2377.

Mease, P., Hall, S., Fitzgerald, O., Van Der Heijde, D., Merola, J.F., Avila-Zapata, F., Cieślak, D., Graham, D., Wang, C., Menon, S., Hendrikx, T., and Kanik, K.S. (2017). Tofacitinib or Adalimumab versus Placebo for Psoriatic Arthritis. *N Engl J Med* 377**,** 1537-1550.

Mease, P.J., Lertratanakul, A., Anderson, J.K., Papp, K., Van Den Bosch, F., Tsuji, S., Dokoupilova, E., Keiserman, M., Wang, X., Zhong, S., Mccaskill, R.M., Zueger, P., Pangan, A.L., and Tillett, W. (2021). Upadacitinib for psoriatic arthritis refractory to biologics: SELECT-PsA 2. *Ann Rheum Dis* 80**,** 312-320.

Panés, J., Sandborn, W.J., Schreiber, S., Sands, B.E., Vermeire, S., D'haens, G., Panaccione, R., Higgins, P.D.R., Colombel, J.F., Feagan, B.G., Chan, G., Moscariello, M., Wang, W., Niezychowski, W., Marren, A., Healey, P., and Maller, E. (2017). Tofacitinib for induction and maintenance therapy of Crohn's disease: results of two phase IIb randomised placebo-controlled trials. *Gut* 66**,** 1049-1059.

Papp, K.A., Menter, M.A., Abe, M., Elewski, B., Feldman, S.R., Gottlieb, A.B., Langley, R., Luger, T., Thaci, D., Buonanno, M., Gupta, P., Proulx, J., Lan, S., and Wolk, R. (2015). Tofacitinib, an oral Janus kinase inhibitor, for the treatment of chronic plaque psoriasis: results from two randomized, placebo-controlled, phase III trials. *Br J Dermatol* 173**,** 949-961.

Sandborn, W.J., Su, C., Sands, B.E., D'haens, G.R., Vermeire, S., Schreiber, S., Danese, S., Feagan, B.G., Reinisch, W., Niezychowski, W., Friedman, G., Lawendy, N., Yu, D., Woodworth, D., Mukherjee, A., Zhang, H., Healey, P., and Panés, J. (2017). Tofacitinib as Induction and Maintenance Therapy for Ulcerative Colitis. *N Engl J Med* 376**,** 1723-1736.

Smolen, J.S., Pangan, A.L., Emery, P., Rigby, W., Tanaka, Y., Vargas, J.I., Zhang, Y., Damjanov, N., Friedman, A., Othman, A.A., Camp, H.S., and Cohen, S. (2019). Upadacitinib as monotherapy in patients with active rheumatoid arthritis and inadequate response to methotrexate (SELECT-MONOTHERAPY): a randomised, placebo-controlled, double-blind phase 3 study. *Lancet* 393**,** 2303-2311.

Takeuchi, T., Tanaka, Y., Iwasaki, M., Ishikura, H., Saeki, S., and Kaneko, Y. (2016). Efficacy and safety of the oral Janus kinase inhibitor peficitinib (ASP015K) monotherapy in patients with moderate to severe rheumatoid arthritis in Japan: a 12-week, randomised, double-blind, placebo-controlled phase IIb study. *Ann Rheum Dis* 75**,** 1057-1064.

Takeuchi, T., Tanaka, Y., Tanaka, S., Kawakami, A., Iwasaki, M., Katayama, K., Rokuda, M., Izutsu, H., Ushijima, S., Kaneko, Y., Shiomi, T., Yamada, E., and Van Der Heijde, D. (2019). Efficacy and safety of peficitinib (ASP015K) in patients with rheumatoid arthritis and an inadequate response to methotrexate: results of a phase III randomised, double-blind, placebo-controlled trial (RAJ4) in Japan. *Ann Rheum Dis* 78**,** 1305-1319.

Tanaka, Y., Takeuchi, T., Yamanaka, H., Nakamura, H., Toyoizumi, S., and Zwillich, S. (2015). Efficacy and safety of tofacitinib as monotherapy in Japanese patients with active rheumatoid arthritis: a 12-week, randomized, phase 2 study. *Mod Rheumatol* 25**,** 514-521.

Taylor, P.C., Keystone, E.C., Van Der Heijde, D., Weinblatt, M.E., Del Carmen Morales, L., Reyes Gonzaga, J., Yakushin, S., Ishii, T., Emoto, K., Beattie, S., Arora, V., Gaich, C., Rooney, T., Schlichting, D., Macias, W.L., De Bono, S., and Tanaka, Y. (2017). Baricitinib versus Placebo or Adalimumab in Rheumatoid Arthritis. *N Engl J Med* 376**,** 652-662.

Van Der Heijde, D., Baraliakos, X., Sieper, J., Deodhar, A., Inman, R.D., Kameda, H., Zeng, X., Sui, Y., Bu, X., Pangan, A.L., Wung, P., and Song, I.H. (2022). Efficacy and safety of upadacitinib for active ankylosing spondylitis refractory to biological therapy: a double-blind, randomised, placebo-controlled phase 3 trial. *Ann Rheum Dis* 81**,** 1515-1523.

Van Der Heijde, D., Deodhar, A., Wei, J.C., Drescher, E., Fleishaker, D., Hendrikx, T., Li, D., Menon, S., and Kanik, K.S. (2017). Tofacitinib in patients with ankylosing spondylitis: a phase II, 16-week, randomised, placebo-controlled, dose-ranging study. *Ann Rheum Dis* 76**,** 1340-1347.

Van Der Heijde, D., Song, I.H., Pangan, A.L., Deodhar, A., Van Den Bosch, F., Maksymowych, W.P., Kim, T.H., Kishimoto, M., Everding, A., Sui, Y., Wang, X., Chu, A.D., and Sieper, J. (2019). Efficacy and safety of upadacitinib in patients with active ankylosing spondylitis (SELECT-AXIS 1): a multicentre, randomised, double-blind, placebo-controlled, phase 2/3 trial. *Lancet* 394**,** 2108-2117.

Van Der Heijde, D., Tanaka, Y., Fleischmann, R., Keystone, E., Kremer, J., Zerbini, C., Cardiel, M.H., Cohen, S., Nash, P., Song, Y.W., Tegzova, D., Wyman, B.T., Gruben, D., Benda, B., Wallenstein, G., Krishnaswami, S., Zwillich, S.H., Bradley, J.D., and Connell, C.A. (2013). Tofacitinib (CP-690,550) in patients with rheumatoid arthritis receiving methotrexate: twelve-month data from a twenty-four-month phase III randomized radiographic study. *Arthritis Rheum* 65**,** 559-570.

Van Vollenhoven, R.F., Fleischmann, R., Cohen, S., Lee, E.B., Garcia Meijide, J.A., Wagner, S., Forejtova, S., Zwillich, S.H., Gruben, D., Koncz, T., Wallenstein, G.V., Krishnaswami, S., Bradley, J.D., and Wilkinson, B. (2012). Tofacitinib or adalimumab versus placebo in rheumatoid arthritis. *N Engl J Med* 367**,** 508-519.

Vermeire, S., Schreiber, S., Petryka, R., Kuehbacher, T., Hebuterne, X., Roblin, X., Klopocka, M., Goldis, A., Wisniewska-Jarosinska, M., Baranovsky, A., Sike, R., Stoyanova, K., Tasset, C., Van Der Aa, A., and Harrison, P. (2017). Clinical remission in patients with moderate-to-severe Crohn's disease treated with filgotinib (the FITZROY study): results from a phase 2, double-blind, randomised, placebo-controlled trial. *Lancet* 389**,** 266-275.

Westhovens, R., Taylor, P.C., Alten, R., Pavlova, D., Enriquez-Sosa, F., Mazur, M., Greenwald, M., Van Der Aa, A., Vanhoutte, F., Tasset, C., and Harrison, P. (2017). Filgotinib (GLPG0634/GS-6034), an oral JAK1 selective inhibitor, is effective in combination with methotrexate (MTX) in patients with active rheumatoid arthritis and insufficient response to MTX: results from a randomised, dose-finding study (DARWIN 1). *Ann Rheum Dis* 76**,** 998-1008.

Zhang, J., Tsai, T.F., Lee, M.G., Zheng, M., Wang, G., Jin, H., Gu, J., Li, R., Liu, Q., Chen, J., Tu, C., Qi, C., Zhu, H., Ports, W.C., and Crook, T. (2017). The efficacy and safety of tofacitinib in Asian patients with moderate to severe chronic plaque psoriasis: A Phase 3, randomized, double-blind, placebo-controlled study. *J Dermatol Sci* 88**,** 36-45.
